# Sestrin2-mediated disassembly of stress granules dampens aerobic glycolysis to overcome glucose starvation

**DOI:** 10.1038/s41420-023-01411-3

**Published:** 2023-04-14

**Authors:** Mingyue Li, Rick Francis Thorne, Ruijie Wang, Leixi Cao, Fangyuan Cheng, Xuedan Sun, Mian Wu, Jianli Ma, Lianxin Liu

**Affiliations:** 1 Department of Hepatobiliary Surgery, The First Affiliated Hospital of USTC, Division of Life Sciences and Medicine, University of Science and Technology of China, 230001 Hefei, Anhui China; 2 Translational Research Institute of People’s Hospital of Zhengzhou University and Academy of Medical Sciences, Zhengzhou University, 450053 Zhengzhou, Henan China; 3 Department of Radiation Oncology, Harbin Medical University Cancer Hospital, 150081 Harbin, Heilongjiang China

**Keywords:** Cell biology, Cancer metabolism

## Abstract

Sestrins are a small gene family of pleiotropic factors whose actions promote cell adaptation to a range of stress conditions. In this report we disclose the selective role of Sestrin2 (SESN2) in dampening aerobic glycolysis to adapt to limiting glucose conditions. Removal of glucose from hepatocellular carcinoma (HCC) cells inhibits glycolysis associated with the downregulation of the rate-limiting glycolytic enzyme hexokinase 2 (HK2). Moreover, the accompanying upregulation of SESN2 through an NRF2/ATF4-dependent mechanism plays a direct role in HK2 regulation by destabilizing HK2 mRNA. We show SESN2 competes with insulin like growth factor 2 mRNA binding protein 3 (IGF2BP3) for binding with the 3′-UTR region of HK2 mRNA. Interactions between IGF2BP3 and HK2 mRNA result in their coalescence into stress granules via liquid-liquid phase separation (LLPS), a process which serves to stabilize HK2 mRNA. Conversely, the enhanced expression and cytoplasmic localization of SESN2 under glucose deprivation conditions favors the downregulation of HK2 levels via decreases in the half-life of HK2 mRNA. The resulting dampening of glucose uptake and glycolytic flux inhibits cell proliferation and protect cells from glucose starvation-induced apoptotic cell death. Collectively, our findings reveal an intrinsic survival mechanism allowing cancer cells to overcome chronic glucose shortages, also providing new mechanistic insights into SESN2 as an RNA-binding protein with a role in reprogramming of cancer cell metabolism.

## Introduction

Sestrins (SESN) comprise a small gene family associated with cellular responses to various stress conditions, including the depletion of nutrients and growth factors along with hypoxia, oxidative stress, ER stress, radiation, and DNA damage [1–6]. They are highly conserved, with the three genes found in vertebrates, *SESN1*, *SESN2* and *SESN3* sharing nearly 50% identity at the amino acid level [7]. The discovery of the founding family member SESN1 showed it was regulated by the tumor-suppressor p53, earning it the alternative name of the p53-activated gene 26 (PA26) and recognition as a key growth arrest and DNA damage-inducible gene (GADD) [8]. A second member, SESN2, also termed the hypoxia-inducible gene 95 (Hi95) was subsequently identified but shown to be regulated independently of p53 [9, 10]. The third sestrin, SESN3, was shown to be activated by the forkhead transcription factor (FoxO) under energy crisis conditions [11, 12]. Collectively, sestrins engage in protective cellular responses against stress while alterations in their function also contributes to various pathologies, including metabolic syndrome diseases such as insulin resistance and lipid accumulation along with aging and cancer [5]. However, among these reports, the role of sestrins in regulating aerobic glycolysis under glucose deprivation stress is poorly understood.

An established hallmark of cancer cells involves their reliance on glycolysis to generate energy even in the presence of oxygen, otherwise called the Warburg effect [13–15]. The first rate-limiting enzyme in aerobic glycolysis involves the conversion of glucose to glucose-6-phosphate (G-6-P) mediated by hexokinases (HK) [16]. Mammalian cells possess four HK isoforms (HK1, HK2, HK3, and HK4) although HK2 is the predominant enzyme expressed by cancer cells including hepatocellular carcinoma (HCC) [17, 18]. Other attributes of HK2 also serve to promote its efficiency in promoting aerobic glycolysis, for example, HK2 binding to the voltage-dependent anion-selective channel protein 1 (VDAC1) activates ATP synthesis-related enzymes to avoid substrate inhibition from G-6-P, thereby enhancing glycolysis [19, 20].

The expression of HK2 is controlled by major signaling pathways including the PI3K/Akt/HIF-1α axis and β-catenin/c-Myc signaling along with the transcription factor STAT3 and miR-199a [21–24]. Other research has also revealed the importance of regulating HK2 mRNA stability which involves interactions with various RNA-binding proteins (RBPs) and RNAs. Of relevance to this report, IGF2BP3 also known as IMP3, is a highly expressed RBP involved in post-transcriptional gene regulation [25–27] which fulfils a variety of mRNA regulatory functions ranging from mRNA stability and degradation, nuclear export and localization along with miRNA biogenesis [28–31]. IGF2BP3 was shown to cooperate with BAG3 to stabilize HK2 mRNA in competition with Roquin to promote aerobic glycolysis [32]. Moreover, different cancer-overexpressed non-coding RNAs also promote HK2 mRNA stability, with circCDKN2B-AS1 complexing with IGF2BP3 and HK2 mRNA to sustain aerobic glycolysis [33], while the related IGF2BP2 protein also increases HK2 mRNA stability through forming a complex with the lncRNA CASC9 [34]. Dual PTEN/p53 deficiency can also stabilize HK2 mRNA through post-transcriptional and translational regulation via inhibition of miR143 biogenesis [35]. However, little is known how the stability of HK2 mRNA is affected by stress responses such as nutrient deprivation conditions.

Intriguingly, IGF2BP3 participates in the formation of ribonucleoprotein (RNP) granules with G3BP and TIAR proteins [36–38]. Alternatively called stress granules, these cytoplasmic structures belong to a class of membraneless organelles formed through liquid-liquid phase separation (LLPS), a process driven through weak multivalent interactions between proteins and nucleic acids [39]. Stress granules are induced under different stress conditions, such as oxidative stress, hypoxia, and nutrient deprivation [38, 40, 41] with the compartmentalization of target mRNAs serving to facilitate mRNA stability [42]. LLPS is also inherently a reversible process allowing the rapid release of their various biomolecular components. Here we reveal a balancing act between SESN2 and IGF2BP3 that contributes to the survival of HCC cells under chronic glucose deprivation conditions. This implicitly involves regulatory effects on HK2 levels where SESN2 impairs the stability of HK2 mRNA with this response competing against IGF2BP3 capturing HK2 mRNA in LLPS-formed stress granules. In this manner, SESN2 suppresses aerobic glycolysis, inhibiting cell proliferation, and preventing cell death through apoptosis. These findings provide new insights into the function of SESN2 in reprogramming glucose metabolism, a mechanism which represents an essential protective response in overcoming the deleterious effects of glucose shortages in cancer cells.

## Results

### Glucose deprivation upregulates SESN2 through NRF2 and ATF4 to promote cell survival

**Fig. 1:**
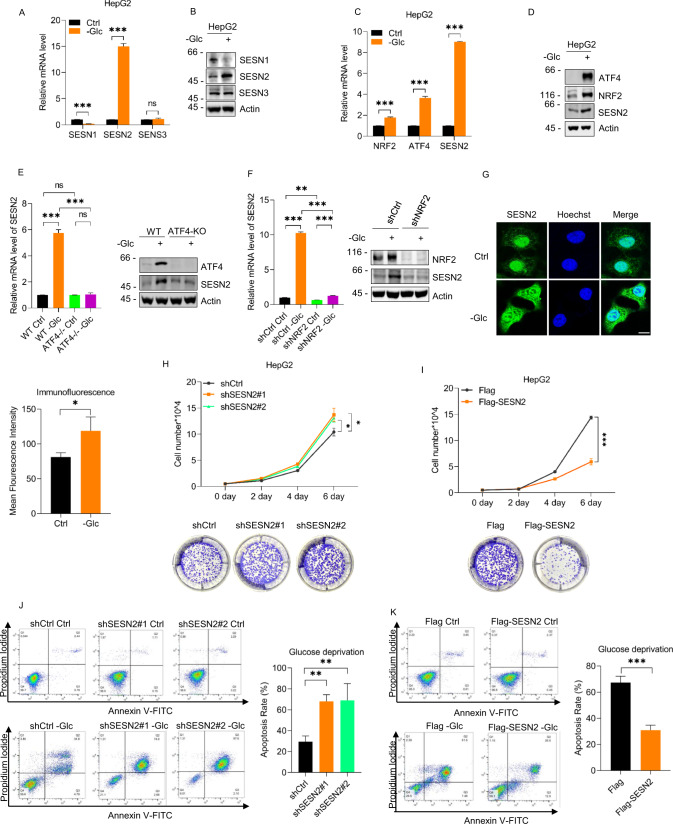
Glucose-deprivation stress upregulates SESN2 through NRF2 and ATF4 to suppress growth and protect cells from apoptosis. **A**, **B** Relative mRNA (**A**) and protein (**B**) levels of SESN1, SESN2, and SESN3 in HepG2 cells determined by qPCR and western blot, respectively, after culture in medium with (+) or without (−) 4 g/L glucose (Glc) for 12 h. Actin was used as a loading control throughout. **C**, **D** Relative mRNA (**C**) and protein (**D**) levels of ATF4, NRF2, and SESN2 in HepG2 cells determined after culture in medium with (+) or without (−) 4 g/L glucose (Glc) for 12 h. **E** Relative mRNA (left) and protein (right) levels of ATF4, NRF2, and SESN2 in WT and ATF4 knockout (KO) HepG2 cells after culture with (+) or without (−) 4 g/L glucose (Glc) for 12 h. **F** Relative mRNA (left) and protein (right) levels of NRF2 and SESN2 in HepG2 cells transduced with control (pLKO.1) or shRNAs targeting NRF2 and cultured in the presence (Ctrl) or absence of glucose (Glc) for 12 h. **G** Representative confocal images of SESN2 immunostaining and MFI (Mean Fluroscence Intensity) in HepG2 cells under control or glucose deprivation conditions for 12 h. Scale bar, 10 μm. **H** Cell growth rates (upper) and colony formation (lower) of HepG2 cells transduced with control (pLKO.1) or shRNAs targeting SESN2 measured as total cell numbers or colony numbers determined over 6 days or 10 days, respectively. **I** Cell growth rates (upper) and colony formation (lower) of HepG2 cells after transfection with Flag control or Flag-SESN2 vectors for 24 h measured as per **H**. **J** Flow cytometric assessment of apoptosis rate using Annexin V-FITC/PI staining in HepG2 cells transduced with control (pLKO.1) or shRNAs targeting SESN2 and cultured in the presence (Ctrl) or absence of glucose (Glc) for 24 h. **K** Annexin V-FITC/PI staining performed in HepG2 cells 24 h after transfection with Flag control or Flag-SESN2 vectors and further cultured in the presence (Ctrl) or absence of glucose (Glc) for 36 hours. **A**–**K** Data represent three independent experiments. **A**, **C**, **E** (left), **F** (left), **H** (upper), **G** (lower), **I** (upper), **J** (right), **K** (right) Data are mean ± SD, *n* = 3, *p < 0.05; **p < 0.01; ***p < 0.001; ns not significant, two-tailed paired Student’s *t* test (**A**, **C**, **I** (upper), **G** (lower), **K** (right)), two-way ANOVA analysis (**E** (left), **F** (left)), one-way ANOVA analysis (**H** (upper), **J** (right)).

Cancer cell survival within the tumor microenvironment relies upon their adaptive responses to range of stresses including hypoxia, acidosis, oxidative stress, and hypernutrition. While SESNs are widely reported to be involved in a range of stress responses, their role in modulating glycolysis during glucose deprivation is poorly understood. Anticipating that SESNs would likely be involved in mounting survival responses against sustained glucose deprivation, we first measured the expression level changes in *SESN1*, *SESN2*, and *SESN3* in the HepG2 HCC cell line. This analysis revealed that SESN2 mRNA and protein levels were significantly increased after glucose withdrawal for 12 h, whereas SESN1 expression was downregulated while SESN3 was unchanged (Fig. 1A, B). To further explore the contribution of the SESNs to glycolysis, each gene was individually silenced in HepG2 cells using shRNAs. Strikingly we observed that depletion of SESN2, but no other sestrin gene increased culture medium acidification and lactate production (Fig. S1A–C), indicating that SESN2 notionally functions as a negative regulator of glycolysis.

It was next important to understand how SESN2 was regulated by glucose deprivation in HCC cells. Taking cues from a previous study reporting SESN2 upregulation in non-small cell lung cancer cells, we investigated the role of the transcription factors ATF4 (activating transcription factor 4) and NRF2 (nuclear factor erythroid 2-related factor 2) [43] in activating SESN2 expression under glucose deprivation in HepG2 cells. Indeed, we discovered that the levels of mRNA and protein of ATF4 and NRF2 were robustly increased in response to glucose deprivation (Fig. 1C, D). Furthermore, knockout of ATF4 and knockdown of NRF2 were each able to prevent the induction of SESN2 mRNA and protein in HepG2 cells deprived of glucose (Fig. 1E, F), indicative that ATF4 and NRF2 cooperate to transactivate SESN2. A further notable finding was made when the subcellular localization of SESN2 was examined using confocal microscopy. Under glucose replete conditions, SENS2 was substantially located in the cell nucleus while glucose withdrawal promoted substantial increases in cytoplasmic staining (Fig. 1G), proposing that changes in SESN2 subcellular localization could be related to its presumed actions in glycolysis inhibition.

Glucose is essential for metabolic homeostasis while prolonged glucose deprivation can induce cell death through apoptosis [44, 45]. Our observations that SESN2 is increased during glucose deprivation while glycolysis is accelerated following SESN2 knockdown suggested that SESN2 functions to dampen glucose utilization when glucose levels are limiting. And given the protective roles of sestrins in overcoming stress conditions, SESN2 is likely to promote cell survival under glucose-deprivation stress. We therefore examined the impact of modulating SESN2 on the proliferation and apoptosis of HepG2 cells. The results of growth and colony formation assays demonstrated that SESN2 knockdown increased proliferation whereas overexpressing SESN2 showed remarkable inhibition (Fig. 1H, I). Further examination of cultures using phase contrast microscopy revealed consistent changes in cell numbers under glucose replete conditions but more intriguing effects in the absence of glucose. In particular, cells with knockdown of SESN2 exhibited substantially more dead and dying (rounded) cells compared with control cells (supplementary Fig. 1D), whereas cells overexpressing SESN2 exhibited lesser numbers of dying cells (supplementary Fig. 1E).

To confirm these data, we performed flow cytometry analyses using Annexin V-FITC/PI staining to quantitate the levels of apoptosis. Instructively, under normal culture conditions, the levels of apoptotic cells were low and not significantly affected by either knockdown or overexpression of SESN2 whereas substantial differences were evident under glucose deprivation. On the one hand, knockdown of SENS2 more than doubled the rates of apoptosis while SENS2 overexpression served to halve the rates (Fig. 1J, K). Supplementing these data, Western blotting showed that glucose deprivation increased the levels of the apoptotic marker cleaved PARP (poly (ADP-ribose) polymerase) in SESN2 knockdown HepG2 cells compared to their control counterparts (supplementary Fig. 1F), while overexpression of SESN2 reduced the levels of cleaved PARP compared with controls (supplementary Fig. 1G). Taken together, these results support our proposition that SESN2 suppresses cell proliferation in response to limiting glucose, preventing apoptosis to enable cell survival. However, the mechanism linking SENS2 to the control of glycolysis remained to be determined.

### SESN2 inhibits glycolysis through reducing the expression of HK2

Our preceding results showed that SESN2 promoted obvious alterations in cell growth and lactate production, changes that we presumed were associated with glycolysis. To formalize this notion, we sought to establish how glucose deprivation affects the glycolytic capacity of HepG2 cells together with the impact of manipulating SENS2.

**Fig. 2:**
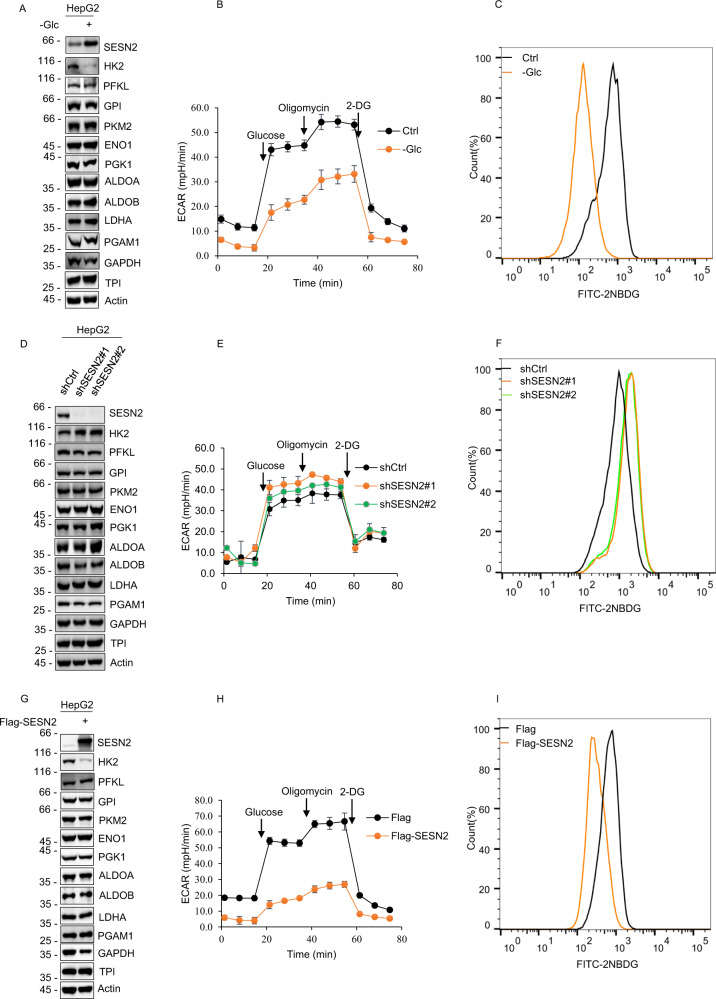
SESN2 inhibits glycolysis via reduction of HK2. **A** Western blot analysis of key glycolytic pathway enzymes along with LDHA and SESN2 in HepG2 cells cultured with (+) or without (−) 4 g/L glucose for 12 h. Actin served as a loading control throughout. **B** Glycolysis stress test profiles measuring the extracellular acidification rate (ECAR) in HepG2 cells after culture in medium with (+) or without (−) 4 g/L glucose (Glc) for 12 h. **C** Glucose uptake measured with 2-NBDG in the cells from **B**. **D** Western blot analysis of key glycolytic pathway enzymes along with LDHA and SESN2 in HepG2 cells after transduction with control (pLKO.1) or independent shRNAs targeting SESN2 (shSESN2#1 and #2, respectively). **E**, **F** Glycolysis stress test profiles (**E**) and glucose uptake (**F**) measured in the cells from **D**. **F** Glucose uptake of HepG2 cells transduced with control (pLKO.1) or shRNAs targeting SESN2 were measured with 2-NBDG. **G** Western blot analysis of key glycolytic pathway enzymes along with LDHA and SESN2 in HepG2 cells 24 hr after transfection with Flag control or Flag-SESN2 vectors. **H**, **I** Glycolysis stress test profiles (**H**) and glucose uptake (**I**) measured in the cells from **G**. **A**–**I** Data represent three independent experiments. Data are mean ± SD, *n* = 3, *p < 0.05; **p < 0.01; ***p < 0.001; ns not significant, two-tailed paired Student’s *t* test.

First, we investigated the impact of glucose deprivation on glycolytic pathway enzymes inclusive of lactate dehydrogenase A (LDHA). However, we observed that glucose deprivation only significantly affected HK2 expression, with robust decreases matching the parallel increases in SESN2 expression (Fig. 2A). As anticipated, measuring the extracellular acidification rate (ECAR), a proxy measure of glycolysis, that glucose deprivation significantly decreased glycolytic flux in HepG2 cells (Fig. 2B). Consistently, 2-NBDG glucose uptake assays measuring glucose consumption showed that glucose uptake capacity was robustly impaired in cells following glucose deprivation (Fig. 2C). We then repeated these assays after SESN2 knockdown or overexpression to determine if there was a causal relationship between SESN2, the levels of HK2 and glycolytic flux. Instructively, the levels of HK2 expression were obviously increased following SESN2 knockdown (Fig. 2D) while HK2 expression was significantly decreased with SESN2 overexpression (Fig. 2G). Moreover, the results of functional assays showed that SESN2 knockdown remarkably enhanced the rates of ECAR and glucose consumption (Fig. 2E, F) compared to SESN2 overexpression which conversely decreased ECAR and glucose consumption (Fig. 2H, I).

Collectively these data propose that SESN2 inhibits HK2 expression resulting in suppression of glucose uptake, glycolytic flux, and lactate production. Nevertheless, the underlying molecular mechanisms involved needed further investigation.

### SESN2 impairs HK2 mRNA stability through competitive interactions between IGF2BP3 and HK2 mRNA

**Fig. 3:**
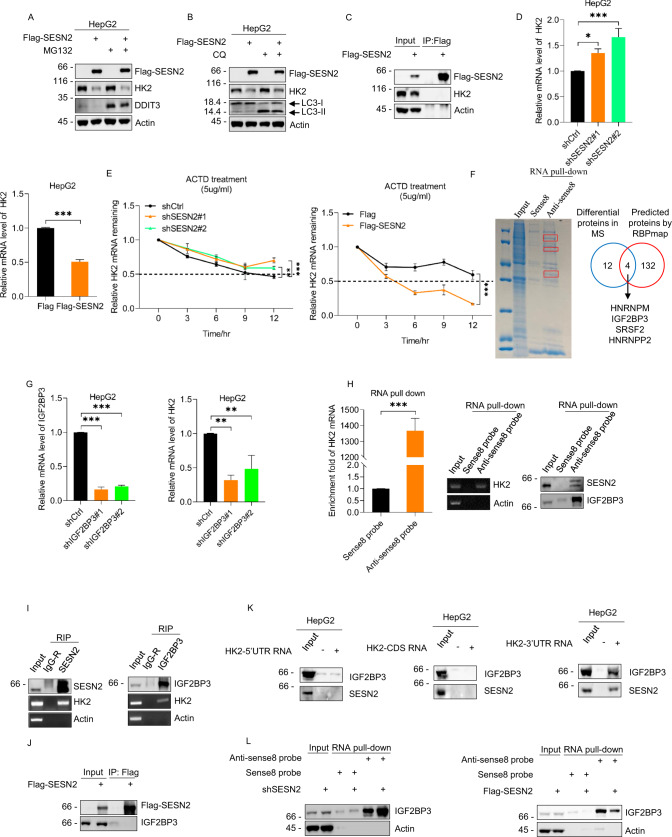
SESN2 impairs the stability of HK2 mRNA via competitive interaction with IGF2BP3. **A** Western blot analysis of SESN2 and HK2 in HepG2 cells 24 h after transfection with Flag control or Flag-SESN2 vectors before treatment with or without 20 μM MG132 for 4 h. Proteasomal inhibition by MG132 treatments was verified by the accumulation of a DDIT3 control with actin used throughout as a loading control. **B** Western blot analysis of SESN2 and HK2 in HepG2 cells 24 h after transfection with Flag control or Flag-SESN2 vectors before treatment with or without 50 μM chloroquine (CQ) for 6 h. Inhibition of lysosomal degradation was verified by the accumulation of LC3-II. **C** Co-immunoprecipitation analyses between SESN2 and HK2. HepG2 cells transfected with Flag control or Flag-SESN2 vectors were pretreated with 20 μM MG132 for 4 h before immunoprecipitation with anti-Flag (M2) antibodies followed by Western blotting against Flag and HK2. **D** qRT-PCR analyses of HK2 mRNA levels in HepG2 cells transduced with control (pLKO.1) or shRNAs targeting SESN2 (top right) or in HepG2 cells 24 h after transfection with Flag control or Flag-SESN2 vectors (bottom left). **E** RNA stability of HK2 mRNA in HepG2 cells transduced with control (pLKO.1) or shRNAs targeting SESN2 (left) or after transfection with Flag control or Flag-SESN2 vectors (right). The indicated cells were treated with 5 μg/ml Actinomycin D (ACTD) for 0, 3, 6, 9, and 12 h before analysis using qRT-PCR. **F** Mass spectrographic identification of proteins associating with HK2 mRNA. Coomassie stained SDS-PAGE gel showing RNA pulldown samples from HepG2 cells captured with biotin-labeled sense (control) and antisense DNA probes targeting HK2 mRNA (left). The red box highlights differentially-recovered proteins (left). Venn diagram illustrating the overlap between 12 proteins identified by MS and HK2 mRNA-interacting proteins predicted from the RBPmap database (http://rbpmap.technion.ac.il/1663329235/results.html) (right). **G** qRT-PCR analyses of IGF2BP3 (left) and HK2 (right) mRNA levels in HepG2 cells transduced with control (pLKO.1) or independent shRNAs targeting IGF2BP3 (shIGF2 P3#1 and #2, respectively). **H** RNA pull-down assays were conducted on HepG2 cells using biotin-labeled sense (control) or antisense DNA probes to capture HK2 mRNA. The recovery of HK2 mRNA was assessed using qRT-PCR (left) and RT-PCR (middle) while the binding of SESN2 and IGF2BP3 was investigated using Western blotting (right). **I** RIP assays were performed in HepG2 cells using antibodies against SESN2 (left) and IGF2BP3 (right). The recovery of SESN2, and IGF2BP3 respectively, was confirmed using Western blotting with the enrichment of HK2 mRNA measured using RT-PCR. **J** HepG2 cells were transfected with either Flag control or Flag-SESN2 vectors and immunoprecipitations performed using anti-Flag (M2) beads. The precipitates were immunoblotted against Flag and IGF2BP3. **K** RNA pulldown assays conducted in HepG2 cells against individual segments of the HK2 mRNA. Probes corresponding to the 5′UTR (left), CDS (middle), and 3′UTR (right) were prepared as in vitro transcribed and biotinylated RNAs with the resulting precipitates subjected to Western blotting against SESN2 and IGF2BP3. **L** HepG2 cells were transduced with control (pLKO.1) or shRNAs targeting SESN2 (left) or transfected for 24 h with Flag control or Flag-SESN2 vectors (right) before conducting RNA pull-down assays against HK2 mRNA as per **H**. Precipitates were immunoblotted for IGF2BP3. **A**–**L** Data represent three independent experiments. **D**, **E**, **G**, **H** Data are mean ± SD, n = 3, *p < 0.05; **p < 0.01; ***p < 0.001; ns not significant, two-tailed paired Student’s *t* test (**D** (right), **E** (right), **H** (left)), one-way ANOVA analysis (**D** (left), **E** (left), **G**).

To determine if the changes in HK2 expression associated with SESN2 were post-translational in nature, we examined the effects of MG132 and chloroquine, inhibitors of proteasomal activity [46] and autophagy, respectively [47], on the levels of HK2 protein. However, neither MG132 nor chloroquine could rescue the reduction in HK2 protein levels in HepG2 cells overexpressing SESN2 (Fig. 3A, B). Moreover, co-immunoprecipitation (co-IP) analyses conducted between ectopically expressed Flag-SESN2 and endogenous HK2 showed that there were no interactions evident between SESN2 and HK2 (Fig. 3C). These data excluded the possibility that SESN2 regulates HK2 expression through post-translational mechanisms.

We turned to consider the alternative hypothesis that HK2 changes involved transcriptional or post-transcriptional mechanisms. Measurement of HK2 mRNA levels using qRT-PCR revealed that knockdown and overexpression of SESN2, respectively, significantly increased and decreased the mRNA levels of HK2 (Fig. 3D). Furthermore, actinomycin D chase assays to examine mRNA stability showed that SESN2 knockdown enhanced HK2 mRNA stability whereas the enforced expression of SESN2 cells significantly decreased HK2 mRNA stability (Fig. 3E). Thus, SESN2 appears to be involved in regulating the stability of HK2 mRNA.

Next to investigate how SESN2 inhibits HK2 mRNA stability, we performed RNA pull-down assays combined with mass spectrometry analysis to interrogate the protein interactome of HK2 mRNA, particularly to look for known RBPs. Comparing results between the biotin-labeled sense DNA probe (control) and antisense (test) probes uncovered 12 proteins selectively recovered with HK2 mRNA. And to further rationalize these data we considered the results of the RBPmap database (http://rbpmap.technion.ac.il/) which predicted 132 RBPs potentially interacting with HK2 mRNA. Intersection of the candidate lists showed only four proteins in common between the between the mass spectrometry analysis and the RBPmap database, namely HNRNPM (Heterogeneous Nuclear Ribonucleoprotein M), FUS (FUS RNA-binding protein), SRSF2 (Serine/arginine-rich splicing factor 2), and IGF2BP3 (Insulin like growth factor 2 mRNA binding protein 3) (Fig. 3F). Of these four proteins, HNRNPM [48–50] and SRSF2 [51] have been shown to be involved in multiple facets of mRNA regulation although FUS has not presently been shown to affect mRNA stability or translation [52]. And as noted in the Introduction, IGF2BP3 has been previously implicated in maintaining HK2 mRNA stability [32–34].

For our initial investigations, we tested if knockdown of any of the four candidate genes resulted in changes to the levels of HK2 mRNA. Notably, knockdown of IGF2BP3 significantly reduced HK2 mRNA levels (Fig. 3G), whereas depleting HNRNPM, FUS, or SRSF2 did not significantly affect HK2 mRNA levels **(**supplementary Fig. 2A–C). Furthermore, Western blotting analysis of the RNA pull-down assays confirmed the interaction between HK2 mRNA and IGF2BP3, proposing a direct role for IGF2BP3 in maintaining HK2 mRNA stability. And surprisingly we also found that SESN2 was present within the HK2 mRNA pulldown samples (Fig. 3H), raising intriguing possibilities as to whether HK2 mRNA associates discretely with IGF2BP3 and SESN2 or otherwise forms a ternary complex.

To resolve these possibilities, we explored interactions between HK2 mRNA, IGF2BP3 and SESN2 using immunoprecipitation. We first confirmed that endogenous HK2 mRNA could be readily recovered in RNA IP (RIP) assays against both SESN2 and IGF2BP3 (Fig. 3I). However, co-immunoprecipitation (co-IP) analyses showed that there were no obvious direct interactions between Flag-SESN2 and IGF2BP3 (Fig. 3J), favoring the notion that SESN2 and IGF2BP3 discretely interact with HK2 mRNA. To decipher which regions of HK2 mRNA were responsible for binding to SESN2 and IGF2BP3, respectively, biotin pull-down assays were performed against in vitro transcribed segments of the HK2 mRNA involving the 5′-UTR, CDS, and 3′-UTR regions. This analysis revealed that both SESN2 and IGF2BP3 were recovered with the 3′-UTR region of HK2 mRNA with no interactions evident with the 5′-UTR and CDS segments (Fig. 3K). Moreover, RNA pull-down assays performed in cells after knockdown or ectopic expression of SESN2 revealed that the amount of IGF2BP3 co-precipitated with HK2 mRNA was increased with SESN2 knockdown but reduced when SESN2 was overexpressed (Fig. 3L). Collectively these findings suggest that SESN2 competes with IGF2BP3 for interactions with the 3′-UTR region of HK2 mRNA, and that this interaction is responsible for impairing the stability of HK2 mRNA.

### SESN2 impedes the formation of IGF2BP3-HK2 mRNA stress granules by impeding phase separation

It has been reported that IGF2BP3 is involved in assembling stress granules, cytoplasmic protein/RNA aggregates in which mRNAs are stored during stress conditions, such as oxidative stress, hypoxia, and nutrient deprivation [38, 40]. This represents a protective response with IGF2BP3-mediated mRNA storage within stress granules representing a key mechanism to enhance mRNA stability [42]. For example, the stability of c-Myc mRNA, a known IGF2BP3 target, was significantly higher in cells with IGF2BP3 overexpression under heat shock conditions [38]. Noting our previous observation that glucose deprivation stress drives the nuclear-cytoplasmic translocation of SENS2, we considered the possibility that SENS2 functions to balance the actions of IGF2BP3 in capturing HK2 mRNA in stress granules.

**Fig. 4:**
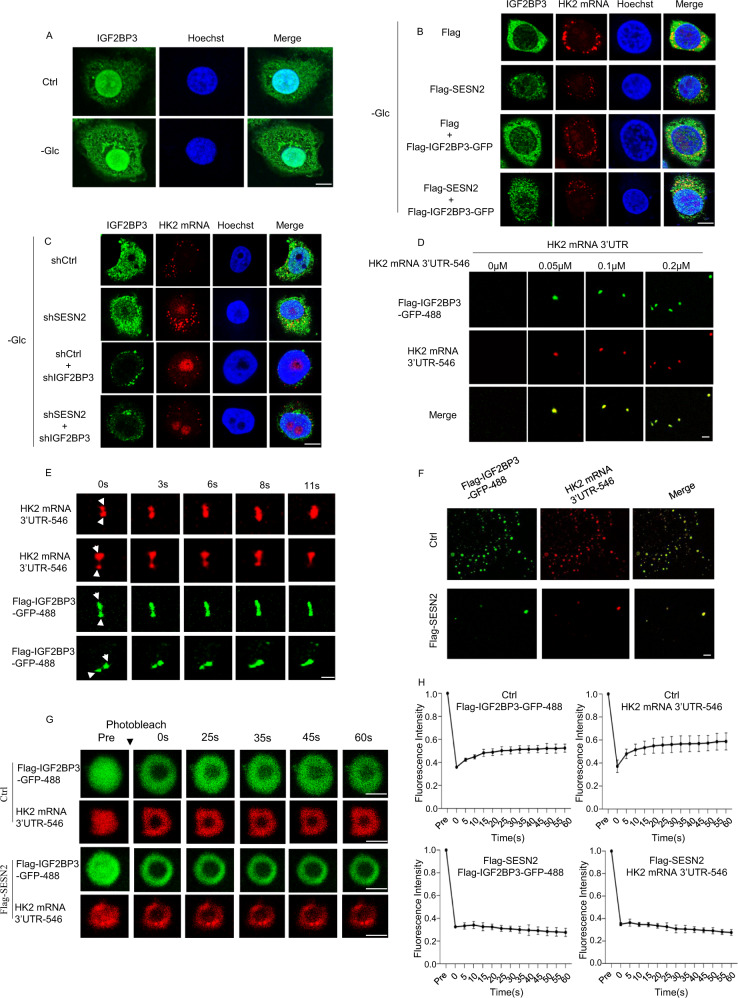
SESN2 impedes the formation of IGF2BP3 and HK2 mRNA-based LLPS droplets. **A** Representative confocal images showing staining of endogenous IGF2BP3 in HepG2 cells under control or glucose deprivation for 12 h conditions. Scale bar, 10 μm. **B** HepG2 cells were transfected for 24 h with the indicated combinations of Flag control, Flag-SESN2, Flag-SESN2 and Flag-IGF2BP3-GFP vectors and further cultured in the absence of glucose (Glc) for 12 hours. The cells were then subjected to combined immunofluorescence-fluorescence in situ hybridization (IF-FISH) with data showing representative confocal images showing staining of IGF2BP3 (green), HK2 mRNA (red) and nuclear counterstaining with Hoechst 33342 (blue). Scale bar, 10μm. **C** HepG2 cells were transduced with control (pLKO.1) or shRNAs targeting SESN2 before culture in the absence of glucose for 12 h. transfected for 24 h with the indicated combinations of Flag control, Flag-SESN2, Flag-SESN2 and Flag-IGF2BP3-GFP vectors and further cultured in the absence of glucose (Glc) for 12 hours. Combinatorial IF-FISH was then conducted as per **B**. Scale bar, 10μm. **E** The formation of liquid-liquid phase separation droplets in vitro was examined by confocal microscopy in solutions of 15 μM purified Flag-IGF2BP3-GFP protein mixed with the indicated concentrations of Alexa Fluor 546-labeled HK2 3′UTR mRNA (0-0.2 μM). Scale bar, 5 μm. **D** Time sequential confocal images observing fusion events between individual liquid-liquid phase separation droplets in a solution of Flag-IGF2BP3-GFP protein (15 μM) and Alexa Fluor 546-labeled HK2 3′UTR mRNA (0.5 μM). Scale bar, 1 μm. **F** The formation of liquid-liquid phase separation droplets in vitro was examined by confocal microscopy in solutions of Flag-IGF2BP3-GFP (15 μM) and Alexa Fluor 546-labeled HK2 mRNA 3′UTR (0.5 μM) in the absence and presence of purified Flag-SESN2 protein (15 μM). Scale bar, 10 μm. **G**, **H** FRAP (fluorescence recovery after photobleaching) assays conducted on liquid-liquid phase separation droplets formed by solutions of Flag-IGF2BP3-GFP (15 μM) and Alexa Fluor 546-labeled HK2 mRNA 3′UTR (0.5 μM) in the absence and presence of purified Flag-SESN2 protein (15 μM). Flag-SESN2 protein was added after droplet formation. Representative pre- and post-bleach confocal microscopy images (scale bar, 1μm) (**G**) with quantification of fluorescence intensity recovery in the bleached region (n = 3 droplets/trace) (**H**). **A**–**H** Data represent three independent experiments. **H** Data are presented as mean ± SD.

We first considered the cellular localization of IGF2BP3 in HepG2 cells. Consistent with the idea that IGF2BP3 drives stress granule formation, confocal imaging showed glucose deprivation resulted in significant increases in IGF2BP3 cytoplasmic puncta (Fig. 4A). To gain further mechanistic insights, we performed immunofluorescence staining combined with RNA fluorescence in situ hybridization (IF-FISH) against IGF2BP3 and HK2 mRNA, respectively, to observe how SESN2 expression affects their subcellular localization. Under glucose deprivation, HK2 mRNA staining predominantly occurred as cytoplasmic granules which partly co-localized with staining with IG2BP3 (Fig. 4B). In this setting, the overexpression of SESN2 remarkably decreased IGF2BP3-HK2 mRNA puncta formation, but it was partially rescued by ectopically expressing Flag-IGF2BP3-GFP. Consistently, IGF2BP3-HK2 mRNA puncta formation was obviously enhanced in cells after SESN2 knockdown while knockdown of IGF2BP3 reversed these changes (Fig. 4C). Together these data suggest that IGF2BP3 captures HK2 mRNA in stress granules whereas SESN2 prevents this process.

As LLPS is the fundamental basis of stress granule formation, we investigated the interactions between IGF2BP3, HK2 mRNA, and SESN2 using in vitro phase separation assays. First, solutions of purified Flag-IGF2BP3-GFP protein were imaged after the addition of different concentrations of in vitro transcribed Alexa Fluor 546-labeled HK2 3′UTR mRNA. Instructively, we observed no droplets were formed with a solution containing only Flag-IGF2BP3-GFP protein whereas the addition of HK2 3′UTR mRNA promoted droplet formation in a dose-dependent manner (Fig. 4D). Moreover, demonstrating an important characteristic of phase separation, smaller droplets formed by Flag-IGF2BP3-GFP were observed to fuse and form larger droplets over time (Fig. 4E). We then assessed the impact of adding purified Flag-SESN2 protein into the reaction system. Notably, the presence of Flag-SESN2 resulted in a marked reduction in the phase separation droplets formed by Flag-IGF2BP3-GFP and Alexa Fluor 546-labeled HK2 mRNA 3′UTR (Fig. 4F). Consistently, fluorescence recovery after photobleaching imaging experiments showed that Flag-SESN2 impeded the dynamic recovery of IGF2BP3 and HK2 mRNA molecules into the photobleached area (Fig. 4G, H).

Taken together, these data suggest that glucose deprivation induces IGF2BP3-mediated stress granule formation via LLPS to capture HK2 mRNA with the further proposition that SESN2 competes for binding to HK2 mRNA to balance this process.

### Competitive interactions between SESN2 and IGF2BP3 regulate glycolysis and cell growth by modulating the expression of HK2

Our preceding data established that glucose deprivation invokes a protective survival mechanism in HepG2 cells via inhibition of glycolysis. This involves the downregulation of HK2 with further analysis showing that competition between SESN2 and IGF2BP3 determines the fate of HK2 mRNA, a mechanism that presumably underlies the effects on HK2 expression and function. To verify this assumption, we examined the outcomes of manipulating SESN2 and IGF2BP3 on HK2 levels, glycolytic regulation and the effects on cell growth.

**Fig. 5:**
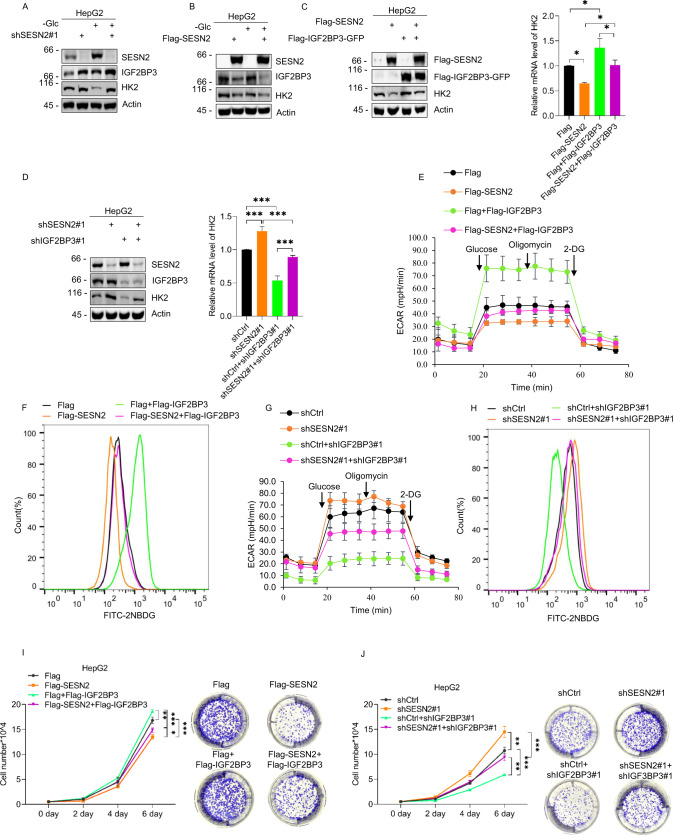
Competition between SESN2 and IGF2BP3 regulates HK2 levels, glycolytic flux, and cell proliferation. **A** Western blot analysis of SESN2, IGF2BP3, and HK2 expression n HepG2 cells after transduction with control (pLKO.1) or independent shRNAs targeting SESN2 comparing cells cultured with (+) or without (−) 4 g/L glucose for 12 h. Actin served as a loading control throughout. **B** Western blot analysis of SESN2 and HK2 expression in HepG2 cells 24 h after transfection with Flag control or Flag-SESN2 vectors before culture with (+) or without (−) 4 g/L glucose for 12 h. **C** HepG2 cells were transfected with the indicated combinations of Flag control, Flag-SESN2 and Flag-IGF2BP3-GFP vectors before determining the expression of HK2 protein and mRNA levels by Western blot (left) and qPCR (right), respectively. Transfection efficiency of SESN2 and IGF2BP3 was confirmed by blotting against anti-Flag. **D** The expression of HK2 protein (left) and mRNA (right) in HepG2 cells transduced with the indicated combinations of control shRNA (pLKO.1) and shRNAs targeting SESN2 and IGF2BP3. **E**, **F** Glycolysis stress test profiles measuring ECAR (**E**) and glucose uptake measured with 2-NBDG (**F**) in HepG2 cells transfected with Flag control, Flag-SESN2, or Flag-IGF2BP3-GFP vectors for 24 h culture in medium with (+) or without (−) 4 g/L glucose (Glc) for 12 h. **G**, **H** Glycolysis stress test profiles measuring ECAR (**G**) and glucose uptake measured with 2-NBDG (**H**) in HepG2 cells transduced with the indicated combinations of control shRNA (pLKO.1) and shRNAs targeting SESN2 and IGF2BP3. **I** Cell growth rates (left) and colony formation (right) of HepG2 cells transfected with the indicated combinations of Flag control, Flag-SESN2 and Flag-IGF2BP3-GFP vectors measured as total cell numbers or colony numbers over 6 days and 10 days, respectively. **J** Cell growth rates (left) and colony formation (right) of HepG2 cells transduced with the indicated combinations of control shRNA (pLKO.1) and shRNAs targeting SESN2 and IGF2BP3 measured as total cell numbers or colony numbers over 6 days and 10 days, respectively. **A**–**J** Data represent three independent experiments. **C** (right), **D** (right), **I** (left), **J** (left) Data are mean ± SD, *n* = 3, *p < 0.05; **p < 0.01; ***p < 0.001; ns not significant, two-way ANOVA analysis.

First, we measured the protein levels of HK2 and IGF2BP3 in response to knockdown or overexpression SESN2 in cells cultured with or without glucose. As expected, removal of glucose increased SESN2 and decreased HK2 levels, respectively while IGF2BP3 expression was also increased to a lesser extent. Moreover, knockdown of SESN2 increased the expression of HK2 and IGF2BP3 under both normal and glucose deprivation conditions (Fig. 5A). Conversely, the overexpression of SENS2 produced opposite effects with the downregulation of HK2 and IGF2BP3 (Fig. 5B). We then compared the effect of manipulating IGF2BP3 to confirm this would antagonize the effects of SENS2 on HK2. Indeed, ectopic expression of IGF2BP2 increased HK2 mRNA levels while the simultaneous overexpression of IGF2BP3 with blunted the SESN2-induced decreases in HK2 mRNA (Fig. 5C). Moreover, individual knockdown of SESN2 and IGF2BP3 resulted in increased and decreased levels of HK2 mRNA and protein, respectively, while their co-knockdown canceled out their singular effects (Fig. 5D).

Lastly, having reconciled that competitive interactions between SESN2 and IGF2BP3 determine the levels of HK2, we examined the resulting cell phenotypes. Measurement of glucose utilization and glycolytic flux showed that the enforced expression of IGF2BP3 promoted glucose uptake and enhanced ECAR whereas the simultaneous overexpression of IGF2BP3 with SESN2 reversed the reductions in glucose uptake and resulting from SESN2 overexpression (Fig. 5E, F). In contrast, knockdown of IGF2BP3 impaired the enhanced glucose uptake and glycolytic flux ECAR resulting from the knockdown of SESN2 (Fig. 5G, H). In accordance with these findings, assessment of cell proliferation using growth curve and colony formation assays showed that overexpression of IGF2BP3 reversed the inhibition of cell proliferation resulting from overexpression of SESN2 (Fig. 5I) while knockdown of IGF2BP3 weakened the growth promoting effects resulting from the knockdown of SESN2 (Fig. 5J). Thus, the balance struck between the functions of SENS2 and IGF2BP3 in regulating HK2 levels imparts tangible effects on cell growth responses.

## Discussion

Recent studies linking SESN2 with the occurrence and development of cancer have highlighted its potential as a diagnostic and therapeutic target [3, 53, 54]. Indeed, SESN2 has been shown to make important contributions to different cancer-related functions including but not limited to roles in regulating cell proliferation, invasion, apoptosis, autophagy, metastasis, and drug resistance [55]. A common thread combining these outcomes involves overcoming various stressors, most notably nutrient starvation where there are clear indications that SESN2 participates in survival responses associated with limiting glucose availability. For instance, the addition of 2-DG to inhibit glycolysis increases SESN2 expression via an AKT-dependent mechanism resulting in the suppression of mTOR signaling [56]. Moreover, SESN2 promotes cell survival by activating PPAR-γ coactivator-1 alpha through the modulation of glutamine metabolism under glucose starvation conditions [57]. Other reports revealed that hypoxia accompanied by glucose starvation upregulates SESN2 to inhibit cell death through necroptosis [43, 57]. However, whether there is any direct role for SESN2 in altering glucose metabolism when glucose is limiting is presently unclear.

**Fig. 6:**
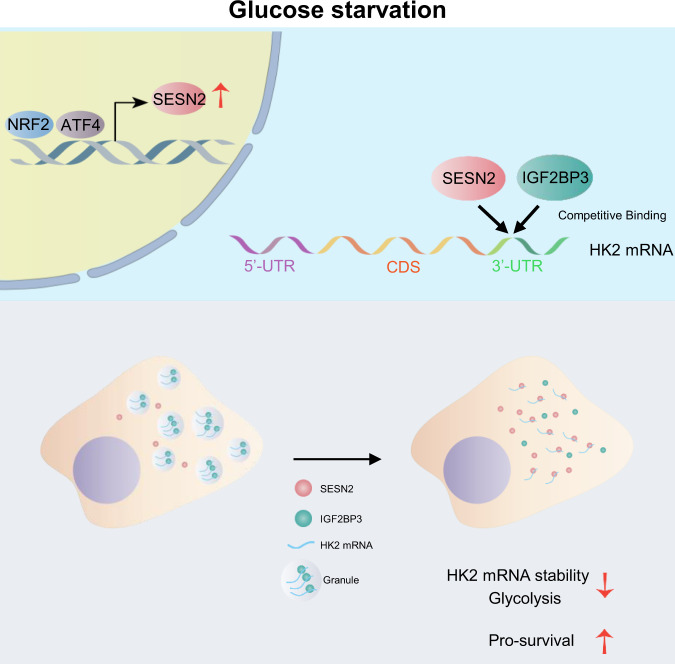
Working model. HK2 mRNA stability is controlled via competitive interactions between SESN2 and IGF2BP3.

Our report now establishes this link, showing that glucose deprivation upregulates SESN2 in HepG2 cells via an ATF4/NRF2-dependent transcriptional mechanism. This served to dampen glycolysis, an effect realized through interplay between SESN2 and IGF2BP3 which determines the fate of HK2 mRNA. On the one hand, we found IGF2BP3 interacts with HK2 mRNA to promote the formation of stress granules, thereby stabilizing HK2 mRNA. On the other hand, SESN2 competes with IGF2BP3 binding, resulting in HK2 mRNA degradation. Notably, glucose deprivation drives the increased expression and cytoplasmic localization of SESN2 to favor the downregulation of HK2, thereby inhibiting glucose utilization and glycolytic flux. The net outcome serves to strike a protective balance, suppressing cell proliferation towards preventing glucose deprivation-induced apoptosis (Fig. 6).

HK2 is the first rate-limiting enzyme in glycolysis [58] and amongst all components of the glycolytic cascade was the only enzyme affected by glucose withdrawal. Genetic manipulation experiments established that SESN2 was involved in the downregulation of HK2 with further work revealing that this involved effects on the post-transcriptional stability of HK2 mRNA through competition between SESN2 and IGF2BP3 for binding to the 3′UTR of HK2 mRNA. The 3′UTR of mRNAs is well known to be a key regulatory region that determines their fate through various interactions with miRNAs and RBPs [29–31]. For example, the recruitment of mRNAs by different stress proteins can prevent their destruction through RISC activity. With regard to IGF2BP3, osmotic stress induces its rapid clustering in the cytoplasm [59], and moreover, IGF2BP3 is among one of many different RBPs known to co-segregate with their mRNA targets to cytoplasmic processing (P) bodies [60]. As noted previously, HK2 mRNA is also a known target of IGF2BP2 and IGF2BP3 [33, 34]. But more remarkable here is the involvement of SESN2 in this paradigm, i.e., we found that SENS2 was the only sestrin induced by glucose deprivation and that its actions as an RBP were essential for modulating the capture and stabilization of HK2 mRNA in stress granules. Indeed, to our best of our knowledge, this is the first study to demonstrate that SESN2 serves as an RNA-binding protein.

As mentioned in the Introduction, stress granules and related membraneless organelles such as P bodies are formed through LLPS [39]. At a fundamental level, these structures enable the rapid condensation of biomolecular components and conversely, their components can also undergo fast dissolution. We found that the RBP activities of IGF2BP3 and SESN2 produced counteractive effects on LLPS to dictate the incorporation of HK2 mRNA into stress granules. Our in vitro investigations showed that HK2 3′UTR mRNA could induce the coalescence of purified IGF2BP3 protein into droplets exhibiting liquid-like properties. The key characteristic features of LLPS [61] observed were (i) that the IGF2BP3 droplets increased in number and size in an RNA-concentration dependent manner, (ii) that discrete droplets could fuse over time, and (iii) that IGF2BP3 and HK2 mRNA were part of the same mobile phase within the droplets. The inclusion of SESN2 in these assays resulted in the patent inhibition of IGF2BP3 droplet formation and moreover, served to disrupt the dynamics of the mobile phase properties of the droplets. While the involvement of IGFBPs in facilitating RNA-protein condensates is not new, there are few reports demonstrating mechanisms that explain how LLPS structures are dispersed. One rare example involves the SARS-CoV-2 nucleocapsid protein phase that serves to disassemble stress granules via interactions with G3BPs [62]. To our best knowledge, the antagonistic actions of SESN2 versus IGF2BP3 constitutes an exemplary observation which could reflect a more widespread phenomenon relevant to many types of membraneless organelles.

Finally, we must consider how this mechanism relates to the persistence of cancer cells within the tumor microenvironment. Cancer cells must evoke protective mechanisms to overcome the effects of a range of deleterious states such as hypoxia, acidosis, and nutrient deficiencies [63]. The strategies adopted to overcome the latter include conserving energy by reducing metabolism and diversifying energy sources to avoid a crisis state that may trigger cell death pathways [64]. In this regard, we found that SESN2 acts as a protective factor to overcome the effects of limiting glucose availability in HCC cells. It is also worth mentioning that it remains controversial as to whether SESN2 principally serves as an oncogene or tumor suppressor gene [55]. Moreover, IGF2BP3 has also been shown to be overexpressed in different cancers and shown to promote cell growth and motility as well as regulating responses to anticancer treatments [42]. Arguably, to pigeon-hole either SESN2 or IGF2BP3 as either as oncogene or tumor suppressor is not overly helpful unless the underlying context is considered. Certainly, the shutdown of proliferation by SESN2 is beneficial for preserving cancer cell vitality but this must be reversed when glucose levels are sufficient to support proliferation. Notwithstanding the need to explore in more detail as to which cancers and genetic subtypes gain advantage from this mechanism, it will be interesting to explore whether other mRNAs associated with different metabolic pathways are also targeted by SESN2.

## Materials and methods

### Cell culture

Cell lines (293 T and HepG2) were routinely cultured in high glucose (25 mM) DMEM (Thermo Fisher Scientific#12800082) or no glucose DMEM (Thermo Fisher#11966025)with 10% v/v fetal bovine serum (BI, Biological Industries#04-001-1ACS), 4mM l-glutamine, 1% v/v penicillin-streptomycin (Gibco#15070063), and 1 mM pyruvate (Gibco#11360070) and maintained at 37 °C in a humidified 5% CO2-containing atmosphere. Cell line authenticity was verified by STR analysis.

### RNA interference and transfections

Gene knockdown experiments were conducted by lentiviral-mediated transduction with short hairpin RNAs (shRNAs). Lentiviral particles were generated by transfection of 293 T cells with PLKO.1 vectors containing specific shRNAs (Table S1) along with pREV, pGag, pVSVG at the ratio of 2:2:2:1 in Opti-MEM medium (Thermo Fisher#31985062) for 48 h. Supernatants were filtered with 0.45μm filter before cells infection, added to target cells for 24 h before selection with 5 μg/ml puromycin (Sigma#P9620). Alternatively, transfections were performed with the indicated plasmids **(**Table S2) using the Lipofectamine-2000 reagent (Invitrogen#11668019) according to the manufacturer’s instructions.

### CRISPR/Cas9-mediated gene deletion

CRISPR/Cas9-mediated ATF4 gene-editing vectors were constructed by annealing gRNA oligonucleotide pairs (Table S3) and subcloning into lentiCRISPRv2 (one vector system) according to the Zhang laboratory protocol. Lentiviral particles produced as described above using a 1:2:2 mixture of plasmids (Pmd2.g, PSPAX2 and lentiCRISPRv2) were used to transduce target cells. After selection with 5 μg/ml puromycin, stably infected cells were plated in 96-well plates and single cell clones screened by Western blot and gDNA sequencing to obtain SESN2 and ATF4 knockout cells.

### Immunofluorescence and single molecule RNA fluorescence in situ hybridization

Cells were grown on glass coverslips before fixation in 4% formaldehyde for 15 min and permeabilization with 0.3% Triton X-100 (Sangon Biotech#A110694-0500) for 10 min at room temperature. For immunofluorescence staining, specimens were blocked with blocking/permeabilizing buffer (1xPBS (Thermo Fisher#21600069), 1% acetylated bovine serum albumin (Sangon Biotech#A600332-0100), 0.3% Triton X-100, 2 mM vanadyl ribonucleoside complexes (Sigma#94740-250MG) in the dark for 60 min at room temperature and washed using 1xPBS before the addition of primary antibodies in blocking/permeabilizing buffer overnight at 4 °C. Specimens were washed and bound antibodies decorated with appropriate fluorochrome-conjugated secondary antibodies diluted in blocking/permeabilizing buffer for 1 h at room temperature. For sequential FISH staining, specimens were washed using 1xPBS and fixed in 4% formaldehyde for 15 min, washed using 1xPBS before to equilibration in wash buffer 2xSSC (Sangon Biotech#B548109-0200), 10% v/v formamide (Diamond#A100314-0100) for 10 min at room temperature. After washing in 1xPBS, the ULS546-labeled HK2-3′UTR-RNA probe was added in hybridization buffer 2xSSC, 10% v/v formamide, 10% dextran sulfate sodium salt (BBI#A600160), RNase inhibitor (Thermo Fisher#B300076) and hybridized overnight at 37 °C. After hybridization, specimens were washed twice for 30 min at 37 °C in wash buffer with Hoechst 33342 (BBI#E607302) nuclear counterstain added during the second wash. Specimens were kept in 2xSSC before mounting the coverslips onto microscope slides using anti-fade agent (Beyotime#P0133) before sealing the coverslips with neutral balsam. Specimens were imaged using a Leica TCS SP8 confocal system.

### Liquid-liquid phase separation

Briefly, the indicated recombinant proteins were diluted in 25 mM Na_2_HPO_4_, 30 mM NaCl, 2 mM DTT (pH 7.4) buffer before mixing with fluor-labeled in vitro transcribed RNA (Ulysis^TM^ Alexa Fluor^TM^ 546; Invitrogen#U21650). The protein-RNA solutions were incubated at room temperature, following which 10 μL of mixture was transferred onto a 35 mm glass bottom dish (NEST 801001) and immediately imaged using a Leica TCS SP8 confocal system with a ×63 oil immersion objective. Confocal images were collected as single Z focal planes focused on the surface of the glass slide. The presence or absence of droplet formation under different conditions (phase separation) was defined by round droplets with diameters greater or equal to 1 μm. All images represent a single focal plane focused onto the surface of the glass slide.

### Fluorescence recovery after photobleaching

FRAP imaging was performed using the FRAP module of the Leica TCS SP8 confocal system with a 63x oil immersion objective. Circular regions of the interest (ROI) were software defined in the phase separation droplets and photobleached using 97% laser power with the 488 nm or 561 nm laser lines as required. Time-series images were acquired every five seconds and the recovery curves from photobleaching was carried out with recorded fluorescence mean intensity. Times-lapse images were collected and fluorescence mean intensity were analyzed with LAS X software.

### RNA stability assay

The half-life of HK2 mRNA was determined by treating cells with Actinomycin D (5 μg/ml, Sigma#SBR00013) over the indicated times (0–12 h). Thereafter, total RNA was isolated with FastPure cell/Tissue Total RNA Isolation Kit V2 (Vazyme#RC112-01) and the levels of HK2 and β-actin determined using qRT-PCR.

### Extracellular acidification rate

Assay were performed using the Seahorse XFe96 analyzer (Seahorse Bioscience, Agilent) according to the manufacturer’s instructions (Seahorse XF Glycolysis Stress Test Kit#103020-100). Cells were seeded at 1×10^4^ cells/well in 96-well XF cell culture micro-plates for 24 h before performing glycolysis stress tests at 37 °C in XF base medium (2 mM glutamine, pH 7.4) with sequential additions of glucose (10 mM), oligomycin (1 μM) and 2-DG (5 mM). Data were analyzed by the Seahorse XF Glycolysis Stress Test Report Generator packages.

### Biotin pull-down assays

For the oligomer-affinity pull-down assays, sense or antisense biotin-labeled DNA oligomers (3ug) corresponding to human HK2 were incubated with streptavidin-coupled Dynabeads (Invitrogen#60210) for 2 h at room temperature. Alternatively, full-length 5′-UTR, CDS, 3′-UTR HK2 products were generated by PCR with the T7 promoter sequence (5′-TAATACGACTCACTATA-3′) incorporated into the 5′ primers **(**Table S3). PCR-amplified DNA was served as the template to transcribe biotinylated RNA using T7 RNA polymerase (Promega#P2077) in the presence of biotin-UTP (Lucigen#ASB71110). Thereafter, 2 μg biotin-labeled RNA was incubated with streptavidin-coupled Dynabeads for 1 hour. Cell lysates from 2–3 × 10^7^ HepG2 cells were incubated with the indicated RNA-protein complexes for 4 h at 4 °C before washing and elution with the resulting samples analyzed by PCR and Western blotting. All processes were performed under RNase-free conditions.

### RNA-IP

Cell lysates were prepared from 2–3 × 10^7^ cells using RIP lysis buffer (0.5% NP-40, 20 mM Tris pH 7.4, 150 mM NaCl, and 1.5 mM MgCl_2_ in DEPC-H_2_O supplemented with protease inhibitor cocktail (Merck#539133)) before incubation with the indicated primary antibodies (Table S6) adsorbed to protein A/G Sepharose beads (Invitrogen#53133) for 4 h at 4 °C. After extensive washing, the bead-bound RNA was extracted and subjected to semi-quantitative RT-PCR using specific primers **(**Table S3). All processes were performed under RNase-free conditions.

### Western blotting and immunoprecipitation

Cellular proteins were extracted using lysis buffer containing 20 mM Tris, 150 mM NaCl, 2 mM EDTA, 1% Triton X-100, and protease inhibitor cocktail and quantified using the BCA protein assay kit (Beyotime#P0012S). Total protein (20 μg/sample) was electrophoresed using SDS-PAGE and transferred to nitrocellulose membranes before blocking with 4% skim milk. Thereafter, the membranes were incubated with primary antibodies overnight at 4 °C followed by horseradish peroxidase-conjugated secondary antibodies with detection using chemiluminescence (Advansta#K-12045-D50). Alternatively, for immunoprecipitations, cell lysates prepared with IP buffer (0.5% NP-40, 20 mM Tris pH 7.4, 150 mM NaCl, 1.5 mM MgCl2, and protease inhibitor cocktail were incubated with primary antibodies adsorbed to protein A/G-Sepharose beads for 4 hours, washed five times with IP buffer. Antibody sources/dilutions are shown in Table S6.

### Metabolite measurements

Extracellular lactate levels and glucose uptake were measured using the Lactate production assay kit (Biovision#K607-100) and 2-NBDG glucose uptake assay kit (Abcam#ab235976), respectively, according to the manufacturer’s instructions.

### Cell growth and colony formation assay

Cells were seeded into 24-well plates at a density of 5,000 cells/well in 500 μl of medium supplemented with 10% FBS and the medium changed every subsequent day. Total cell numbers were counted at the indicated times using a Count star automated cell counter. Alternatively, colony formation assays were initiated by seeding cells into flat-bottomed 6-well plates at a density of 1000 cells/well in 2 ml of medium supplemented with 10% FBS. The medium was changed every subsequent day. After 10 days, cell colonies were washed with PBS and fixed in 4% formaldehyde for 15 min at room temperature before and staining with 1% crystal violet staining solution for 10 min. Background staining was removed by rinsing the wells with PBS.

### Annexin V-FITC/PI apoptosis assay

The rates of apoptosis were detected using the Annexin V-FITC/PI staining kit (BestBio#BB-4101) according to the manufacturer’s procedures. Briefly, cells grown in 6-well plates were harvested, washed with cold PBS and re-suspended in 1× Annexin-binding buffer at °C before the sequential addition of 5 μl Annexin V-FITC solution for 15 min and then 10 μl PI for 5 min. The cells were analyzed using flow cytometry within 1 h of staining.

### Statistical information

Data were assumed to be normally distributed and continuous variables expressed as mean ± SD. All analyses were performed by two-tailed Student’s t-test, one-way ANOVA analysis or two-way ANOVA analysis using GraphPad Prism 8 with significance defined as *p* ≤ 0.05 (ns, not significant, *P < 0.05, **P < 0.01, ***P < 0.001). Reproducibility and the number of replicates used are defined in the corresponding Figure legends.

## Supplementary information

Fig.S1

Fig.S2

Supplementary Figure legends

Supplementary Tables

Original western-blotting data

## Data Availability

Data is available from the corresponding authors upon reasonable request.
