# Supplementary figures and images for: Sestrin2-mediated disassembly of stress granules dampens aerobic glycolysis to overcome glucose starvation
Source: Cell Death Discov. 2023 Apr 14;9:127. doi: 10.1038/s41420-023-01411-3 (PMC10103035; doi:10.1038/s41420-023-01411-3)

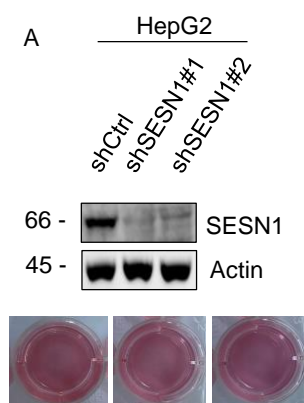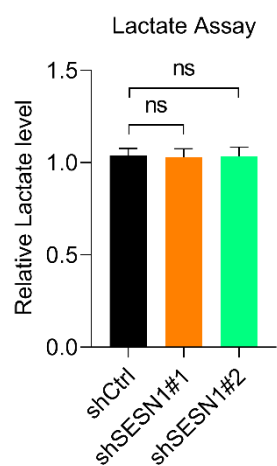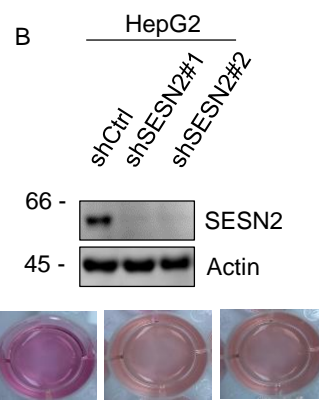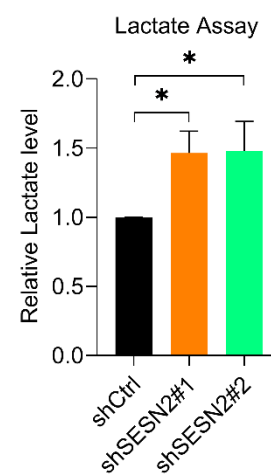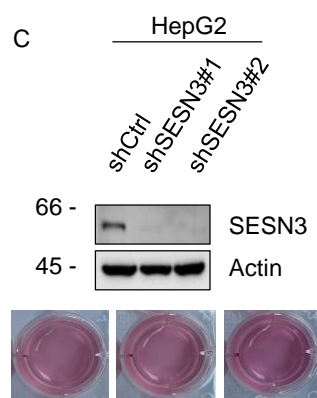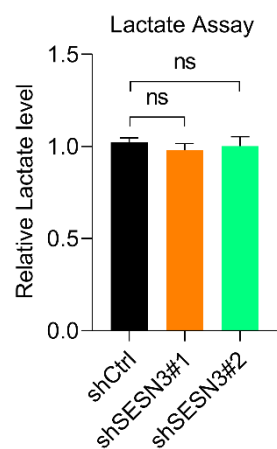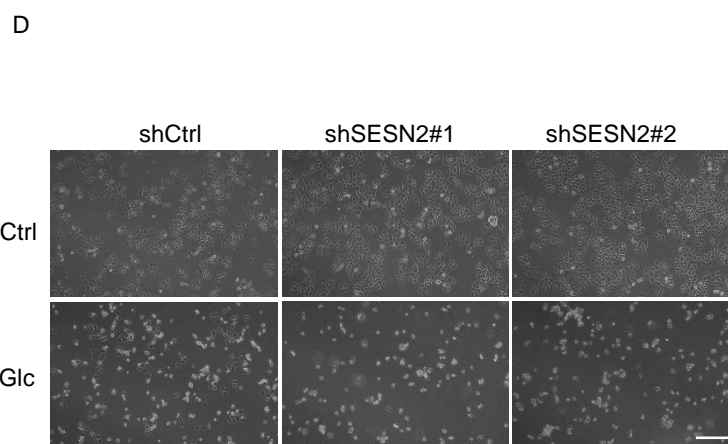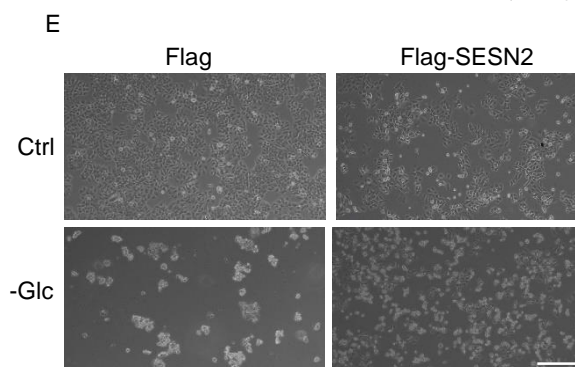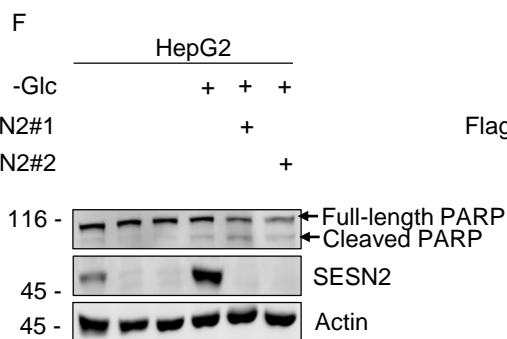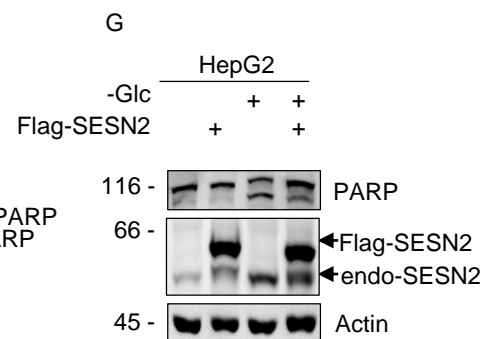

Supplement: Supplementary file 1 — Fig.S1 [file 41420_2023_1411_MOESM1_ESM.pdf]

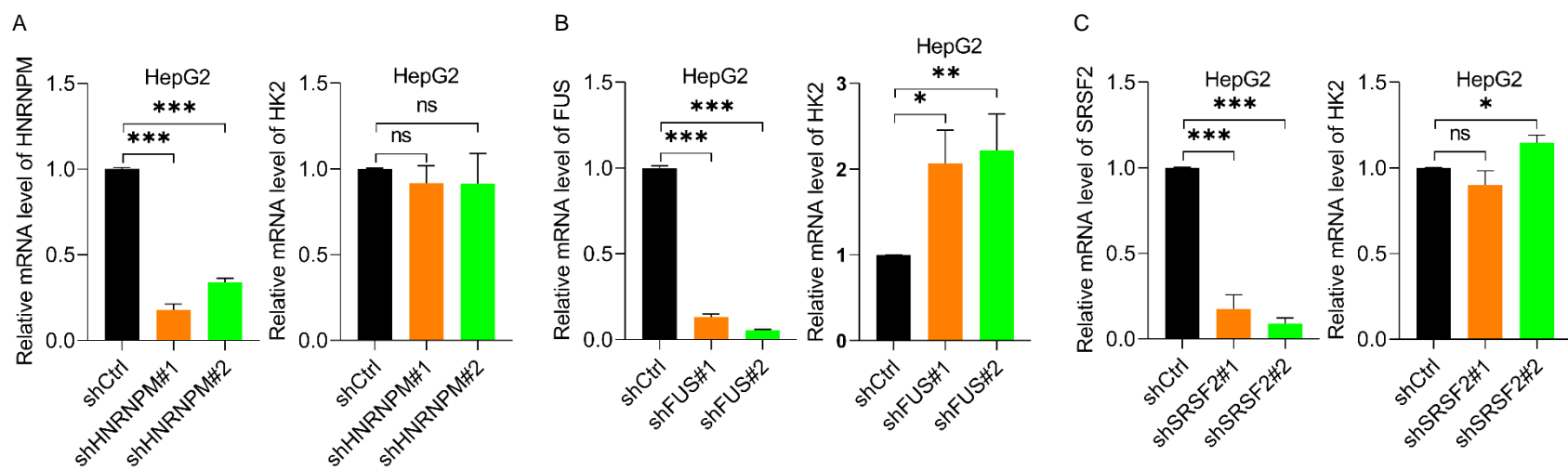

Supplement: Supplementary file 2 — Fig.S2 [file 41420_2023_1411_MOESM2_ESM.pdf]
