# Supplementary material for: Sestrin2-mediated disassembly of stress granules dampens aerobic glycolysis to overcome glucose starvation
Source: Cell Death Discov. 2023 Apr 14;9:127. doi: 10.1038/s41420-023-01411-3 (PMC10103035; doi:10.1038/s41420-023-01411-3)
Supplement: Supplementary file 3 — Supplementary Figure legends [file 41420_2023_1411_MOESM3_ESM.docx]

## Supplementary Fig. 1: SESN2, not SESN1 or SESN3 significantly inhibits lactate production

**(A-C)** HepG2 cells were transduced with empty pLKO.1 vector (shCtrl) or two independent shRNAs targeting SESN1 (A), SENS2 (B), and SESN3 (C) before assessing the efficiency of knockdown using Western blotting (upper panels). Actin was used throughout as a loading control. The extent of culture medium acidification was then compared at 72h (lower panels) along with lactate production (right).

**(D)** Representative phase-contrast micrographs of HepG2 cells transduced with control (pLKO.1) or shRNAs targeting SESN2 after culture with (Ctrl) or without glucose (Glc) for 24 hours. Scale bar, 100μm.

**(E)** Representative phase-contrast micrographs of HepG2 cells transfected with Flag control or Flag-SESN2 overexpression vectors after culture with (Ctrl) or without glucose (Glc) for 36 hours. Scale bar, 100μm.

**(F)** Western blotting of full-length PARP and cleaved-PARP and SESN2 in HepG2 cells transduced with control (pLKO.1) or shRNAs targeting SESN2 after culture with (Ctrl) or without glucose (Glc) for 24 hours.

**(G)** Western blotting of full-length PARP and cleaved-PARPand SESN2 in HepG2 cells transfected with Flag control or Flag-SESN2 vectors after culture in the presence (Ctrl) or absence of glucose (Glc) for 36 hours.

**(A-C** (left), **D-G)** Data represent three independent experiments. **A-C** (right), show mean ± SD, n=3, *p<0.05; **p<0.01; ***p<0.001; ns, not significant, one-way ANOVA analysis.

## Supplementary Fig. 2: Supporting data related to Figure 3

**(A-C)** HepG2 cells were transduced with control (pLKO.1) or independent shRNAs targeting HNRNPM **(A)**, FUS **(B)** or SRSF2 **(C)** before conducting qRT-PCR analyses of HNRNPM, FUS or SRSF2 mRNA (left) and HK2 mRNA (right) levels.

**(A-C)** Data represent three independent experiments. Data are mean ± SD, n=3, *p<0.05; **p<0.01; ***p<0.001; ns, not significant, one-way ANOVA analysis.
