# Supplementary material for: Sestrin2-mediated disassembly of stress granules dampens aerobic glycolysis to overcome glucose starvation
Source: Cell Death Discov. 2023 Apr 14;9:127. doi: 10.1038/s41420-023-01411-3 (PMC10103035; doi:10.1038/s41420-023-01411-3)
Supplement: Supplementary file 4 — Supplementary Tables [file 41420_2023_1411_MOESM4_ESM.docx]

# Table S1. Plasmids used for gene knockdown

| Name | Resistance marker | Vector |
| --- | --- | --- |
| shCtrl | Ampicillin | pLKO.1 |
| shSESN1(#1) | Ampicillin | pLKO.1 |
| shSESN1(#2) | Ampicillin | pLKO.1 |
| shSESN2(#1) | Ampicillin | pLKO.1 |
| shSESN2(#2) | Ampicillin | pLKO.1 |
| shSESN3(#1) | Ampicillin | pLKO.1 |
| shSESN3(#2) | Ampicillin | pLKO.1 |
| shNRF2(#1) | Ampicillin | pLKO.1 |
| shNRF2(#2) | Ampicillin | pLKO.1 |
| shHNRNPM(#1) | Ampicillin | pLKO.1 |
| shHNRNPM(#2) | Ampicillin | pLKO.1 |
| shFUS(#1) | Ampicillin | pLKO.1 |
| shFUS(#2) | Ampicillin | pLKO.1 |
| shSRSF2(#1) | Ampicillin | pLKO.1 |
| shSRSF2(#2) | Ampicillin | pLKO.1 |
| shIGF2BP3(#1) | Ampicillin | pLKO.1 |
| shIGF2BP3(#2) | Ampicillin | pLKO.1 |
| CRISPR-Cas9-KO ATF4(clone1-2) | Ampicillin | lentiCRISPR V2 |
| Rev | Ampicillin | Rev |
| Gag | Ampicillin | Gag |
| VSV-G | Ampicillin | VSV-G |
| psPAX2 | Ampicillin | psPAX2 |
| pMD2.G | Ampicillin | pMD2.G |

# Table S2. Plasmids used for gene transfection

| Name | Resistance marker | Vector |
| --- | --- | --- |
| Flag | Ampicillin | pSin-3Xflag |
| Flag-SESN2 | Ampicillin | pSin-3Xflag |
| Flag-SESN2-GFP | Ampicillin | pSin-3Xflag |
| Flag-IGF2BP3-GFP | Ampicillin | pSin-3Xflag |

# Table S3. Primers Information

| Usage | Name | Primer Sequence 5’-3’ |
| --- | --- | --- |
| Knockdown genes | shSESN1(#1) | CCGGCCAGGACCAATGGTAGACAAACTCGAGTTTGTCTACCATTGGTCCTGGTTTTTTG |
| Knockdown genes | shSESN1(#2) | CCGGGCAAAGAATGGGACTTGGATACTCGAGTATCCAAGTCCCATTCTTTGCTTTTTTG |
| Knockdown genes | shSESN2(#1) | CCGGGCGGAACCTCAAGGTCTATATCTCGAGATATAGACCTTGAGGTTCCGCTTTTTTG |
| Knockdown genes | shSESN2(#2) | CCGGGAAGACCCTACTTTCGGATATCTCGAGATATCCGAAAGTAGGGTCTTCTTTTTTG |
| Knockdown genes | shSESN3(#1) | CCGGCAGTTCTCTAGTGTCAAAGTTCTCGAGAACTTTGACACTAGAGAACTGTTTTTTG |
| Knockdown genes | shSESN3(#2) | CCGGGCTATCCTGAGAGAACTACAACTCGAGTTGTAGTTCTCTCAGGATAGCTTTTTTG |
| Knockdown genes | shNRF2(#1) | CCGGGCTCCTACTGTGATGTGAAATCTCGAGATTTCACATCACAGTAGGAGCTTTTT |
| Knockdown genes | shNRF2(#2) | CCGGGCACCTTATATCTCGAAGTTTCTCGAGAAACTTCGAGATATAAGGTGCTTTTT |
| Knockdown genes | shHNRNPM(#1) | CCGGAGAGCCTTCATTACAAACATACTCGAGTATGTTTGTAATGAAGGCTCTTTTTT |
| Knockdown genes | shHNRNPM(#2) | CCGGACAAGCATAGTCTGAGCGGAACTCGAGTTCCGCTCAGACTATGCTTGTTTTTT |
| Knockdown genes | shFUS(#1) | CCGGCGGACTATGTAATTGTAACTACTCGAGTAGTTACAATTACATAGTCCGTTTTT |
| Knockdown genes | shFUS(#2) | CCGGCGTGGTGGCTTCAATAAATTTCTCGAGAAATTTATTGAAGCCACCACGTTTTT |
| Knockdown genes | shSRSF2(#1) | CCGGGGCAAGCAGTGTAAACGGAGGCTCGAGCCTCCGTTTACACTGCTTGCCTTTTT |
| Knockdown genes | shSRSF2(#2) | CCGGTATCGGCAAGCAGTGTAAACGCTCGAGCGTTTACACTGCTTGCCGATATTTTT |
| Knockdown genes | shIGF2BP3(#1) | CCGGCGGTGAATGAACTTCAGAATTCTCGAGAATTCTGAAGTTCATTCACCGTTTTTG |
| Knockdown genes | shIGF2BP3(#2) | CCGGGCAGGAATTGACGCTGTATAACTCGAGTTATACAGCGTCAATTCCTGCTTTTTG |
| CRISPR-Cas9 construction | ATF4-oligo1-F | CACCGAATGAGCTTCCTGAGCAGCG |
| CRISPR-Cas9 construction | ATF4-oligo1-R | AAACCGCTGCTCAGGAAGCTCATTC |
| CRISPR-Cas9 construction | ATF4-oligo2-F | CACCGATCACAAGTGTCATCCAACG |
| CRISPR-Cas9 construction | ATF4-oligo2-R | AAACCGTTGGATGACACTTGTGATC |
| QRT-PCR | QRT-Actin-F | GACCTGACTGACTACCTCATGAAGAT |
| QRT-PCR | QRT-Actin-R | GTCACACTTCATGATGGAGTTGAAGG |
| QRT-PCR | QRT-SESN1-F | ACAGGGACCAAGCAGATTCA |
| QRT-PCR | QRT-SESN1-R | GGCCCATCCATTTGCAGTAG |
| QRT-PCR | QRT-SESN2-F | CGCAAACTCAGCGAGATCAA |
| QRT-PCR | QRT-SESN2-R | CAGGACCAGAGCCTGAATGA |
| QRT-PCR | QRT-SESN3-F | GCCTGAACTGGTACATGCTG |
| QRT-PCR | QRT-SESN3-R | TTGCTGCCTGAAAGAGATGC |
| QRT-PCR | QRT-ATF4-R | AGGTGTTCTCTGTGGGTCTG |
| QRT-PCR | QRT-ATF4-R | GTACCTAGTGGCTGCTGTCT |
| QRT-PCR | QRT-NRF2-R | CGCAGACATTCCCGTTTGTA |
| QRT-PCR | QRT-NRF2-R | AGCAATGAAGACTGGGCTCT |
| QRT-PCR | QRT-HK2-R | GACGAGAGCATCCTCCTCAA |
| QRT-PCR | QRT-HK2-R | GTTCACCACAGCAACCACAT |
| Plasmid construction | SESN2-PCR-F | cgGAATTCATGATCGTGGCGGACTCCGAGTGCCG |
| Plasmid construction | SESN2-PCR-R | ggACTAGTTCAGGTCATGTAGCGGGTGATGGCAC |
| Plasmid construction | SESN2-GFP-PCR-F | CACCCGCTACATGACCGTGAGCAAGGGCGAGGAGCTGTTCAC |
| Plasmid construction | SESN2-GFP-PCR-R | CATGCGGATCCTTCGACTACTTGTACAGCTCGTCCATGCCGAGAG |
| Plasmid construction | IGF2BP3-PCR-F | CGATGACGACAAGATGAACAAACTGTATATCGGAAACCT |
| Plasmid construction | IGF2BP3-PCR-R | AGCTCCTCGCCCTTGCTCACCTTCCGTCTTGACTGAGGTGGTC |
| Plasmid construction | IGF2BP3-GFP-PCR-F | GTGAGCAAGGGCGAGGAGCT |
| Plasmid construction | IGF2BP3-GFP-PCR-F | CATGCGGATCCTTCGACTACTTGTACAGCTCGTCCATGCCGAGAG |
| RNA pull-down | Sense7 probe | CCGTAACATTCTCATCGATTTC |
| RNA pull-down | Antisense7 probe | GAAATCGATGAGAATGTTACGG |
| RNA pull-down | Sense8 probe | ACTGAGTTTGACCAGGAGATT |
| RNA pull-down | Antisense8 probe | AATCTCCTGGTCAAACTCAGT |
| RNA pull-down | T7-5’UTR-F | TAATACGACTCACTATACGGCCGCGCCCGCGAGCCGTGAGCGA |
| RNA pull-down | T7-5’UTR-R | CCTGCCGCGGCCCGACGGCGCAGAGT |
| RNA pull-down | T7-CDS-F | TAATACGACTCACTATAATGATTGCCTCGCATCTGCTTGCCTA |
| RNA pull-down | T7-CDS-R | CTATCGCTGTCCAGCCTCACGGATGC |
| RNA pull-down | T7-3’UTR-F | TAATACGACTCACTATAAACCCCTGAAATCGGAAGGGACTTCCTCTTTCT |
| RNA pull-down | T7-3’UTR-R | TTTGATTATTTTGGAAAATGTTAAAATTTATTAATAATAGTTA |
| FISH | T7-Probe7-F | TAATACGACTCACTATATGCAGCAGGGCCAGGCAGTCAC |
| FISH | T7-Probe7-R | TGATGTGGCTGTGGATGAGCTTTCAC |
| FISH | T7-Probe8-F | TAATACGACTCACTATATGTTGAGAAGCTCTGGGCTGAG |
| FISH | T7-Probe8-R | ACATCGACATGGTGGAAGGCGATGAG |
| Phase Separation | 546-T7-3’UTR-F | TAATACGACTCACTATAAACCCCTGAAATCGGAAGGGACTTCCTCTTTCT |
| Phase Separation | 546-T7-3’UTR-R | TTTGATTATTTTGGAAAATGTTAAAATTTATTAATAATAGTTA |

# Table S4. Top 12 proteins exhibiting differential binding to HK2 mRNA identified in the mass spectrographic analyses from Figure 3F.

| Description | ∑Unique peptides | MW [kDa] |
| --- | --- | --- |
| HNRNPM | 8 | 77.5 |
| PTBP1 | 6 | 56.5 |
| MTREX | 4 | 117.7 |
| HSPA1L | 4 | 70.3 |
| SRSF2 | 3 | 35.0 |
| FUS | 2 | 75.0 |
| IGF2BP3 | 1 | 63.7 |
| HRNR | 1 | 282.2 |
| FLNA | 1 | 245.7 |
| TXN | 1 | 11.7 |
| ZCCHC8 | 1 | 79.0 |
| CIRBP | 1 | 19.0 |

# Table S5. HK2 mRNA binding proteins predicted using the RBPmap database

| Gene name | Gene name | Gene name |
| --- | --- | --- |
| A1CF | IGF2BP2 | RBM41 |
| ANKHD1 | IGF2BP3 | RBM42 |
| BOLL | ILF2 | RBM45 |
| BRUNOL4 | KHDRBS1 | RBM46 |
| BRUNOL5 | KHDRBS2 | RBM47 |
| BRUNOL6 | KHDRBS3 | RBM4B |
| CELF1 | KHSRP | RBM5 |
| CNOT4 | LIN28A | RBM6 |
| CPEB1 | MATR3 | RBM8A |
| CPEB2 | MBNL1 | RBMS1 |
| CPEB4 | MSI1 | RBMS2 |
| DAZ3 | NOVA1 | RBMS3 |
| DAZAP1 | NUPL2 | RC3H1 |
| EIF4G2 | PABPC1 | SAMD4A |
| ELAVL4 | PABPC3 | SART3 |
| ENOX1 | PABPC4 | SF1 |
| ESRP1 | PABPC5 | SFPQ |
| ESRP2 | PABPN1 | SNRNP70 |
| EWSR1 | PABPN1L | SNRPA |
| FMR1 | PCBP1 | SRSF1 |
| FUBP1 | PCBP2 | SRSF10 |
| FUBP3 | PCBP3 | SRSF11 |
| FUS | PCBP4 | SRSF2 |
| FXR1 | PPRC1 | SRSF4 |
| FXR2 | PRR3 | SRSF5 |
| G3BP2 | PTB3 | SRSF7 |
| HNRNPA0 | PTBP3 | SRSF8 |
| HNRNPA1 | PUF60 | SRSF9 |
| HNRNPA1L2 | PUM1 | TAF15 |
| HNRNPA2B1 | PUM2 | TARDBP |
| HNRNPC | QKI | TIA1 |
| HNRNPCL1 | RALY | TRA2A |
| HNRNPD | RBFOX1 | TRNAU1AP |
| HNRNPDL | RBFOX2 | TUT1 |
| HNRNPF | RBFOX3 | U2AF2 |
| HNRNPH1 | RBM15B | UNK |
| HNRNPH2 | RBM22 | YBX1 |
| HNRNPK | RBM23 | YBX2 |
| HNRNPL | RBM24 | ZC3H10 |
| HNRNPM | RBM25 | ZC3H14 |
| HNRNPU | RBM28 | ZCRB1 |
| HNRPLL | RBM3 | ZFP36 |
| HuR | RBM38 | ZNF326 |
| IGF2BP1 | RBM4 | ZNF638 |

# Table S6. Antibody information

| Antibody | Source | Dilutions |
| --- | --- | --- |
| anti-SESN1 | Proteintech#21668-1-AP | WB 1:1000 |
| anti-SESN2 | Proteintech#21346-1-AP | WB 1:1000  IF 1:100 |
| anti-SESN3 | Proteintech#11431-2-AP | WB 1:1000 |
| anti-NRF2 | Proteintech#16396-1-AP | WB 1:1000 |
| anti-IGF2BP3 | Proteintech#14642-1-AP | WB 1:1000  IF 1:100 |
| anti-HK2 | Cell Signaling Technology#2106 | WB 1:1000 |
| anti-PFKL | Cell Signaling Technology#13029 | WB 1:1000 |
| anti-GPI | Cell Signaling Technology#57893 | WB 1:1000 |
| anti-ALDOA | Cell Signaling Technology#3188 | WB 1:1000 |
| anti-ALDOB | Proteintech#18065-1-AP | WB 1:1000 |
| anti-PGK1 | Cell Signaling Technology#68540 | WB 1:1000 |
| anti-PKM2 | Cell Signaling Technology#3198 | WB 1:1000 |
| anti-PGAM1 | Cell Signaling Technology#12098 | WB 1:1000 |
| anti-ENO1 | Cell Signaling Technology#3810 | WB 1:1000 |
| anti-LDHA | Cell Signaling Technology#2012 | WB 1:1000 |
| anti-TPI | Proteintech#10713-1-AP | WB 1:1000 |
| anti-ATF4 | Proteintech#10833-1-AP | WB 1:1000 |
| anti-PARP | Cell Signaling Technology#9532 | WB 1:1000 |
| anti-mouse IgG(H+L) | Cell Signaling Technology#7076 | WB 1:5000 |
| anti-rabbit IgG(H+L) | Cell Signaling Technology#7074 | WB 1:5000 |
| anti-FLAG | Sigma-Aldrich#F7425 | WB 1:2000 |
| Donkey anti-Rabbit IgG Alexa Fluor 488 | Invitrogen#A-21206 | IF 1:500 |
| anti-GAPDH | CMC-TAG#AT0002 | WB 1:2000 |
| anti-Actin | CMC-TAG#AT0001 | WB 1:2000 |
