# Supplementary material for: Sestrin2-mediated disassembly of stress granules dampens aerobic glycolysis to overcome glucose starvation
Source: Cell Death Discov. 2023 Apr 14;9:127. doi: 10.1038/s41420-023-01411-3 (PMC10103035; doi:10.1038/s41420-023-01411-3)
Supplement: Supplementary file 5 — Original western-blotting data [file 41420_2023_1411_MOESM5_ESM.pdf]

Fig.1

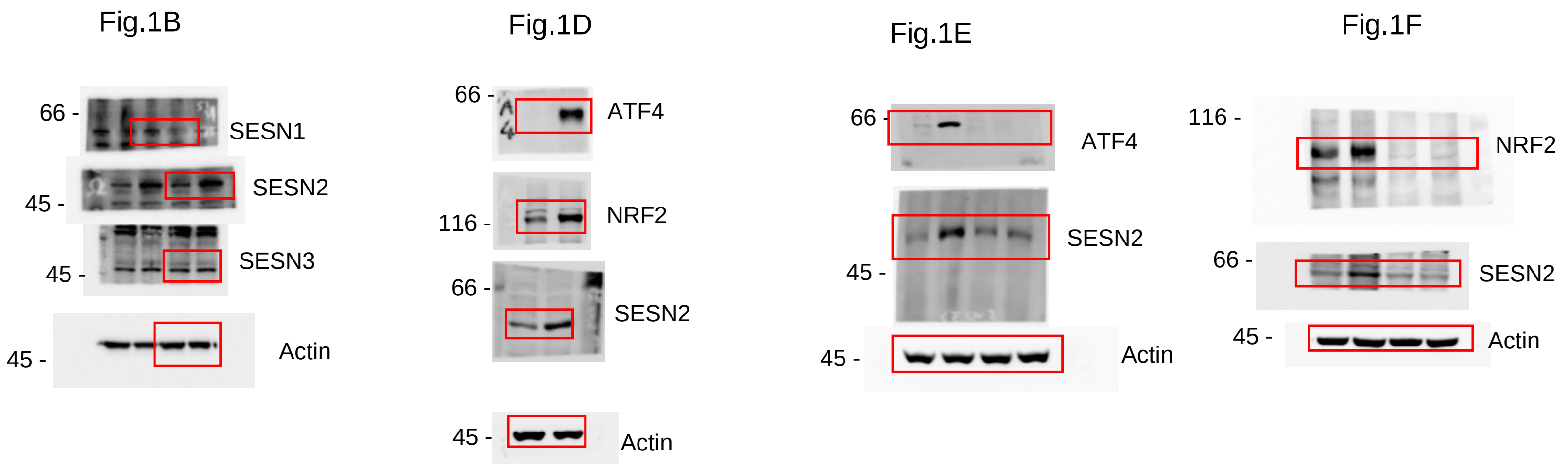
Fig.1B
66 -
SESN1
45 -
SESN2
45 -
SESN3
45 -
Actin
Fig.1D
66 -
ATF4
116 -
NRF2
66 -
SESN2
45 -
Actin
Fig.1E
66 -
ATF4
SESN2
45 -
45 -
Actin
Fig.1F
116 -
NRF2
66 -
SESN2
45 -
Actin

Fig.S1

Fig.S1A

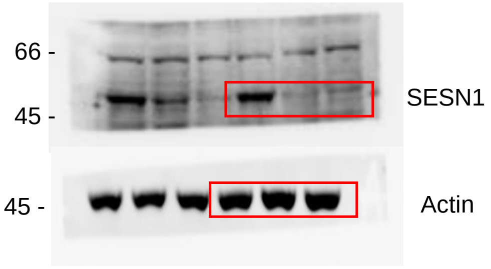

Fig.S1B

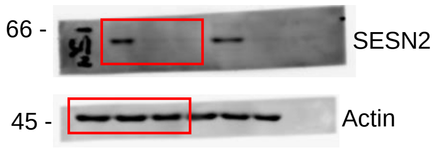

Fig.S1C

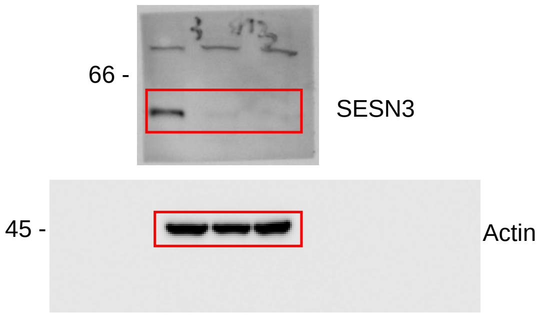

Fig.S1F

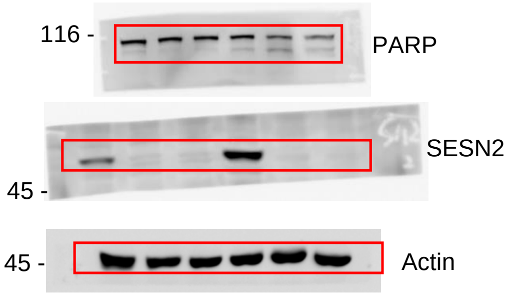

Fig.S1G

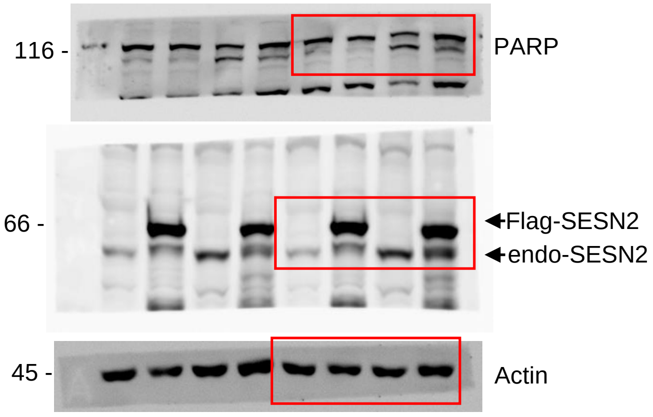

Fig.2

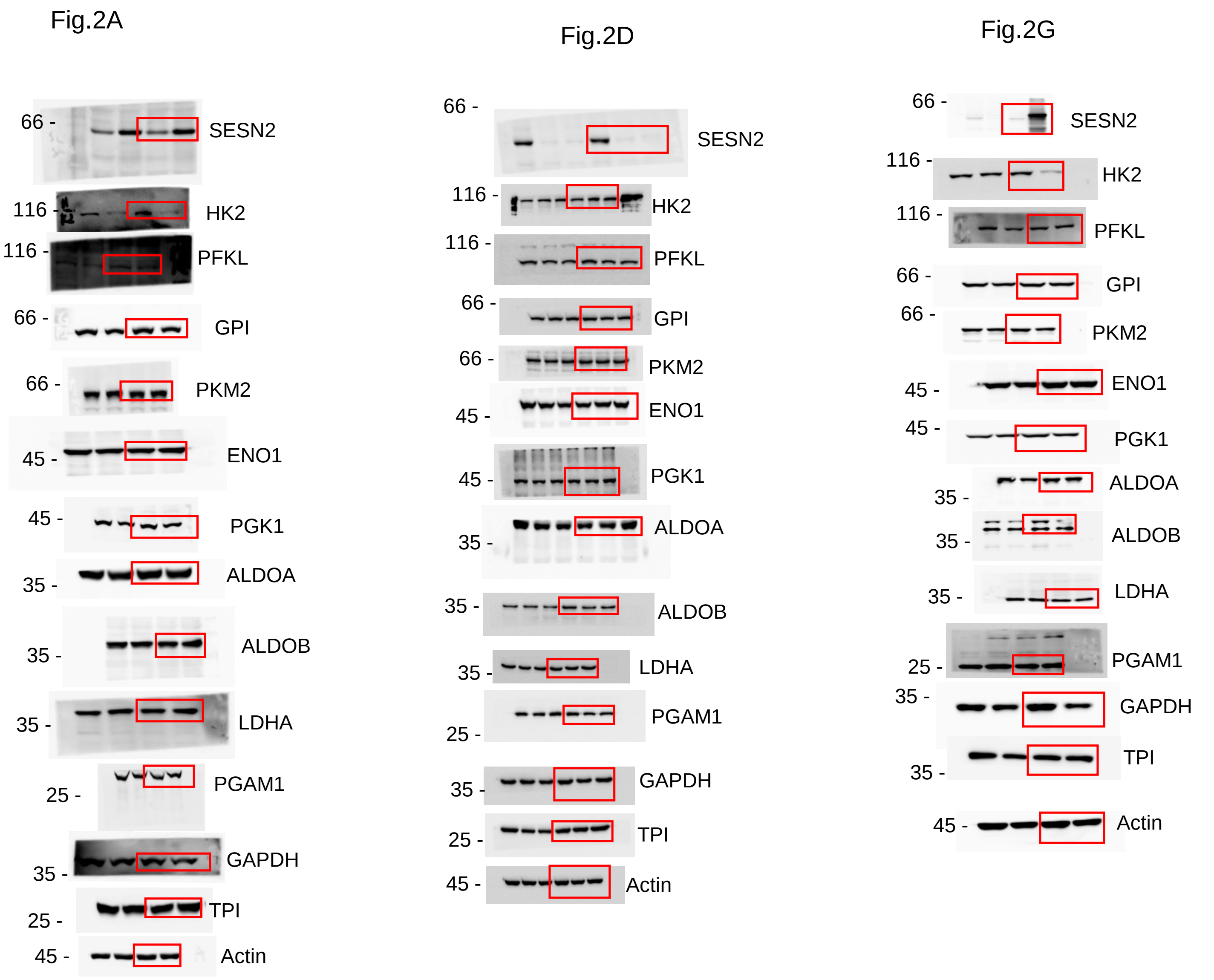

Fig.2A
66 -
SESN2
116 -
HK2
116 -
PFKL
66 -
GPI
66 -
PKM2
45 -
ENO1
45 -
PGK1
35 -
ALDOA
35 -
ALDOB
35 -
LDHA
25 -
PGAM1
35 -
GAPDH
25 -
TPI
45 -
Actin
Fig.2D
66 -
SESN2
116 -
HK2
116 -
PFKL
66 -
GPI
66 -
PKM2
45 -
ENO1
45 -
PGK1
35 -
ALDOA
35 -
ALDOB
35 -
LDHA
25 -
PGAM1
35 -
GAPDH
25 -
TPI
45 -
Actin
Fig.2G
66 -
SESN2
116 -
HK2
116 -
PFKL
66 -
GPI
66 -
PKM2
45 -
ENO1
45 -
PGK1
35 -
ALDOA
35 -
ALDOB
35 -
LDHA
25 -
PGAM1
35 -
GAPDH
35 -
TPI
45 -
Actin

Fig.3

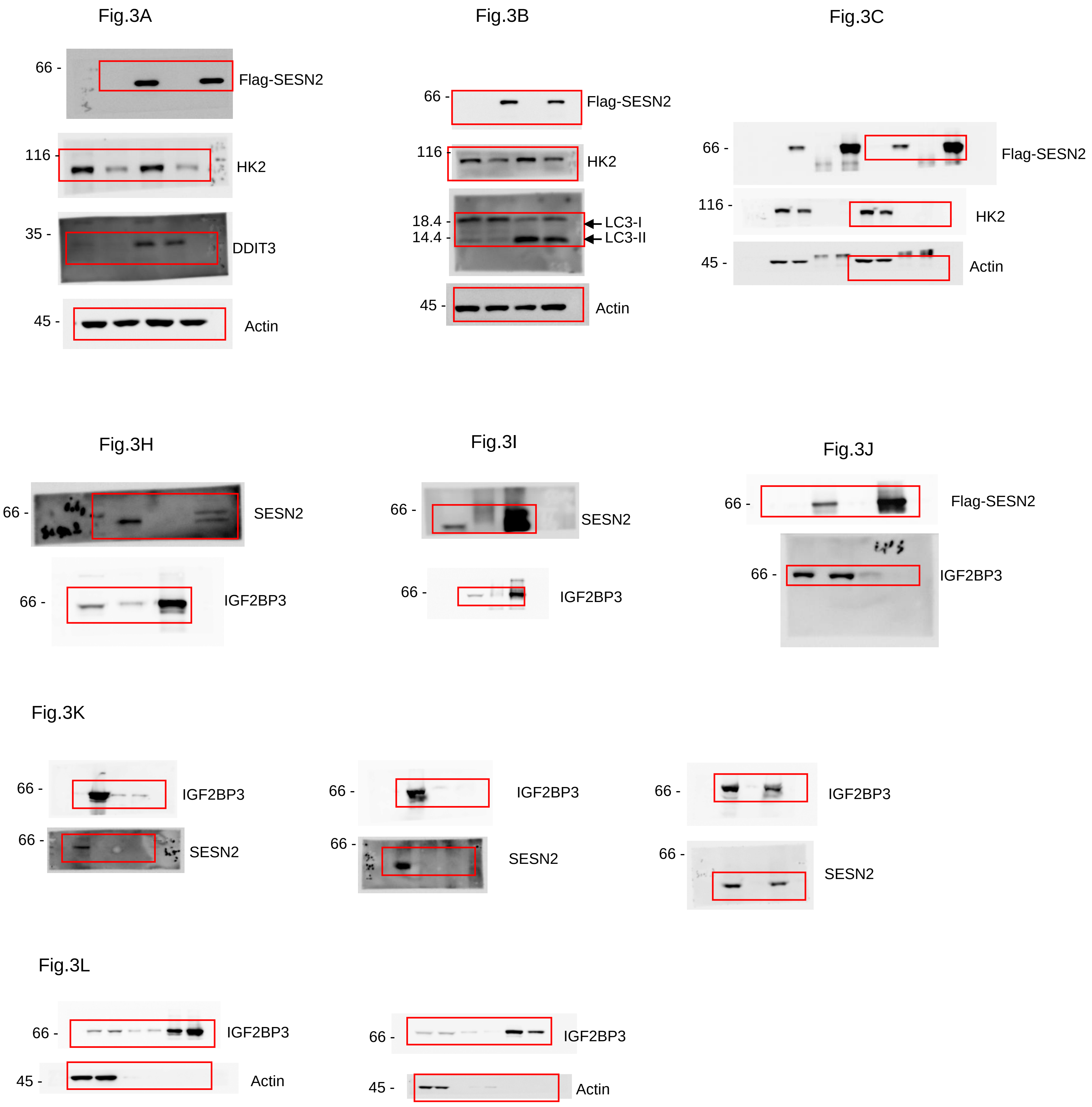
Fig.3A
66 -
Flag-SESN2
116 -
HK2
35 -
DDIT3
45 -
Actin
Fig.3B
66 -
Flag-SESN2
116 -
HK2
18.4 -
14.4 -
LC3-I
LC3-II
45 -
Actin
Fig.3C
66 -
Flag-SESN2
116 -
HK2
45 -
Actin
Fig.3H
66 -
SESN2
66 -
IGF2BP3
Fig.3I
66 -
SESN2
66 -
IGF2BP3
Fig.3J
66 -
Flag-SESN2
66 -
IGF2BP3
Fig.3K
66 -
IGF2BP3
66 -
SESN2
66 -
IGF2BP3
66 -
SESN2
66 -
IGF2BP3
66 -
SESN2
Fig.3L
66 -
IGF2BP3
45 -
Actin
66 -
IGF2BP3
45 -
Actin

Fig.5

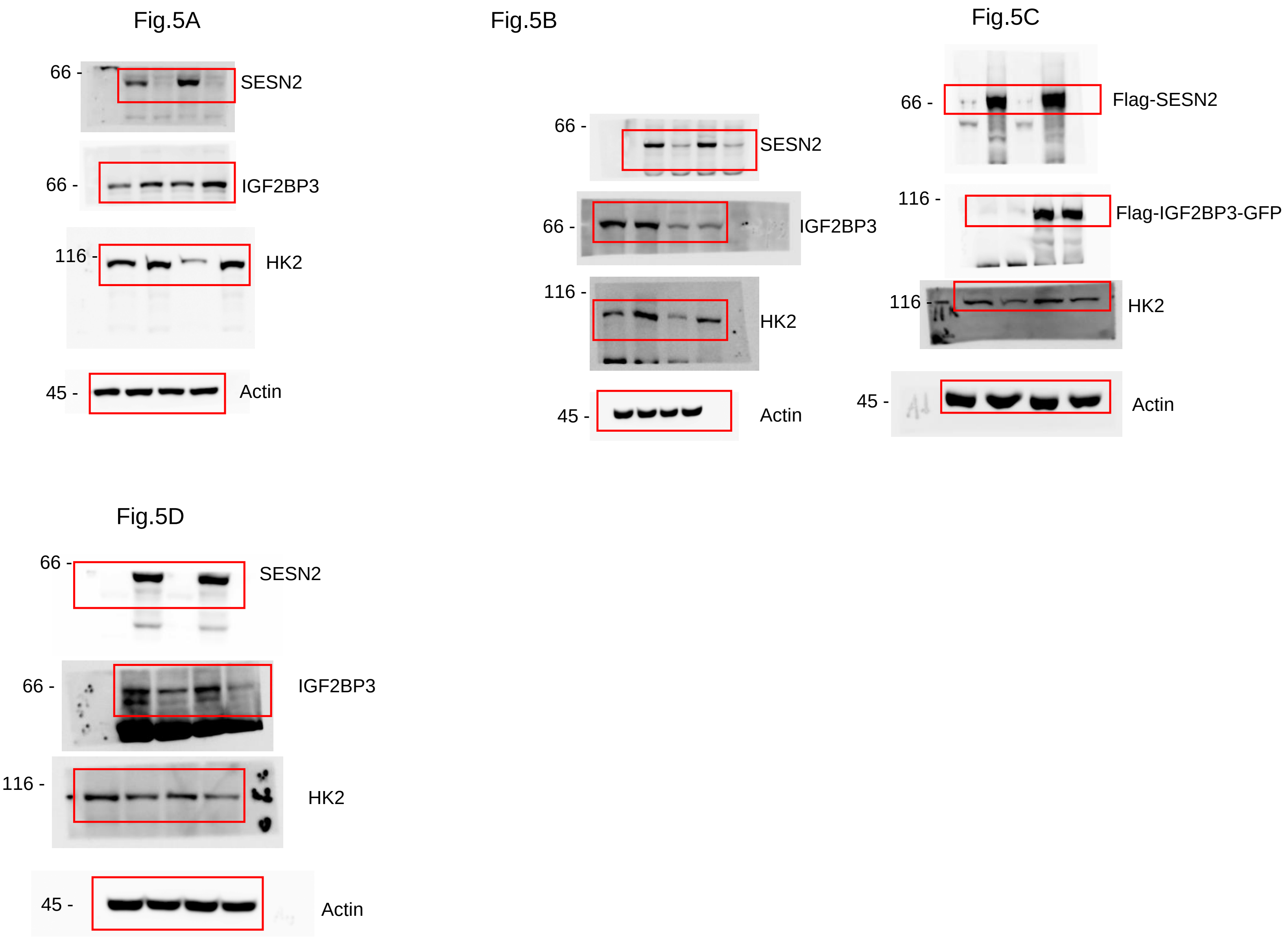
Fig.5A
66 -
SESN2
66 -
IGF2BP3
116 -
HK2
45 -
Actin
Fig.5B
66 -
SESN2
66 -
IGF2BP3
116 -
HK2
45 -
Actin
Fig.5C
66 -
Flag-SESN2
116 -
Flag-IGF2BP3-GFP
116 -
HK2
45 -
Actin
Fig.5D
66 -
SESN2
66 -
IGF2BP3
116 -
HK2
45 -
Actin
